# Identification of synergistic drug combinations using breast cancer patient-derived xenografts

**DOI:** 10.1038/s41598-020-58438-0

**Published:** 2020-01-30

**Authors:** Tia H. Turner, Mohammad A. Alzubi, J. Chuck Harrell

**Affiliations:** 1 Department of Pathology, Virginia Commonwealth University, Richmond, VA USA; 2 Wright Center for Clinical and Translational Research, Virginia Commonwealth University, Richmond, VA USA; 3 Integrative Life Sciences Doctoral Program, Virginia Commonwealth University, Richmond, VA USA; 4 Massey Cancer Center, Virginia Commonwealth University, Richmond, VA USA

**Keywords:** Breast cancer, Breast cancer

## Abstract

Compared with other breast cancer subtypes, triple-negative breast cancer (TNBC) is associated with relatively poor outcomes due to its metastatic propensity, frequent failure to respond to chemotherapy, and lack of alternative, targeted treatment options, despite decades of major research efforts. Our studies sought to identify promising targeted therapeutic candidates for TNBC through *in vitro* screening of 1,363 drugs in patient-derived xenograft (PDX) models. Using this approach, we generated a dataset that can be used to assess and compare responses of various breast cancer PDXs to many different drugs. Through a series of further drug screening assays and two-drug combination testing, we identified that the combination of afatinib (epidermal growth factor receptor (EGFR) inhibitor) and YM155 (inhibitor of baculoviral inhibitor of apoptosis repeat-containing 5 (BIRC5; survivin) expression) is synergistically cytotoxic across multiple models of basal-like TNBC and reduces PDX mammary tumor growth *in vivo*. We found that YM155 reduces EGFR expression in TNBC cells, shedding light on its potential mechanism of synergism with afatinib. Both EGFR and BIRC5 are highly expressed in basal-like PDXs, cell lines, and patients, and high expression of both genes reduces metastasis-free survival, suggesting that co-targeting of these proteins holds promise for potential clinical success in TNBC.

## Introduction

In 2019, it is estimated that over 268,000 American women will be diagnosed with invasive breast cancer, and over 41,000 will have fatal outcomes from the disease^1,2^. Survival rates have considerably improved over the past several decades due to the identification and characterization of distinct histologic and molecular subtypes of breast cancer^3–6^, which predict patient outcomes and have led to the development of targeted therapeutics, allowing treatment regimens to be tailored based on specific tumor characteristics^7–10^. Estrogen receptor (ER)/progesterone receptor (PR) positive (predominantly luminal) tumors or human epidermal growth factor receptor 2 (HER2) positive (HER2-enriched) tumors, which collectively make up the majority of breast cancer cases, are treated with ER- or HER2-targeted drugs, respectively, largely contributing to the current overall breast cancer 5-year survival rate of nearly 90%^1,2^. However, for the approximately 15% of breast cancers that are histologically triple-negative, no clinically successful targeted therapies have yet been developed, despite major translational research efforts^11^. Patients with triple-negative breast cancer (TNBC), a relatively aggressive and highly metastatic subtype, are therefore limited to treatment with chemotherapy, which is highly toxic and often ineffective in treating advanced disease, leading to relatively poor outcomes compared to patients with other subtypes of breast cancer^12–14^. Development of successful therapeutic strategies for TNBC is a challenge due not only to the current lack of reliable drug targets, but also to the heterogeneity of the disease; TNBC can be classified based on gene expression profiles into six distinct subtypes, each of which is dominated by distinct molecular pathways, contributing to differential responses to chemotherapy and investigational targeted therapies^15,16^. Nearly 60% of TNBCs are basal-like, which is characterized by a high propensity to metastasize to vital organs and is associated with a particularly poor prognosis^7,9,10^. In the realm of translational breast cancer research, there is a critical need to identify reliable molecular targets in each subtype of TNBC, particularly basal-like, to enable the development of tailored therapeutic regimens with superior efficacy and less toxicity than current standard-of-care chemotherapeutic cocktails.

In the present study, we performed *in vitro* screening of 1,363 drugs in ten breast cancer patient-derived xenograft (PDX) models, which are known to faithfully recapitulate the characteristics of human disease^17–21^ and are therefore suitable models for studying tumor biology and drug response, both *in vivo* and *ex vivo*/*in vitro*^22–35^. Using this approach, we have generated a dataset that can be used to quickly assess and compare responses of breast cancer PDXs of varying subtypes to many different drugs, most of which are approved by the U.S. Food and Drug Administration (FDA) for various cancer or non-cancer indications. From these data, we identified 176 drugs that were consistently effective across four basal-like TNBC PDXs, encompassing a wide variety of molecular targets and mechanisms of action. Several of these drugs have shown promising efficacy in TNBC and other solid tumors, however it is likely that incorporation into combination regimens is needed to maximize their efficacy and thus their likelihood of clinical success. Through a series of *in vitro* drug response assays, we selected four drugs to test in various two-drug combinations: carfilzomib (proteasome inhibitor), afatinib (epidermal growth factor receptor (EGFR) inhibitor), and YM155 (inhibitor of baculoviral inhibitor of apoptosis repeat-containing 5 (BIRC5; survivin) expression), along with carboplatin, a chemotherapeutic that is part of the current standard-of-care for TNBC and that we have previously tested in several PDXs^36^. Of the six drug combinations tested, we found that the combination of afatinib and YM155 was synergistically cytotoxic across four basal-like TNBC PDXs, and this drug combination significantly reduced PDX mammary tumor growth *in vivo*, without observable toxicity. Notably, the genes encoding the targets of these drugs (EGFR and BIRC5) were found to be highly expressed across basal-like PDXs, cell lines, and patients. Furthermore, our analyses demonstrated that high co-expression of EGFR and BIRC5 in patients was associated with reduced metastasis-free survival, suggesting that co-targeting of EGFR and BIRC5 may be a promising strategy for effective treatment of advanced basal-like TNBC. Herein, we also provide preliminary insight into a potential mechanism of synergism between afatinib and YM155 in the context of this disease.

Collectively, these studies provide valuable insights into PDX drug screening, as well as resources for studying breast cancer drug response profiles, which can inform further drug development studies. Through systematic *in vitro* screening assays, we have uncovered a synergistic combination that, to our knowledge, has not yet been established or explored in TNBC. After further investigation, the combination of afatinib and YM155, and other therapeutic regimens that may be developed based on the data generated in these studies, can potentially make rapid translational impacts on treatment decisions and outcomes for TNBC patients.

## Results

### Drug screening of breast cancer PDXs reveals potential targeted therapeutic candidates for TNBC

**Figure 1:**
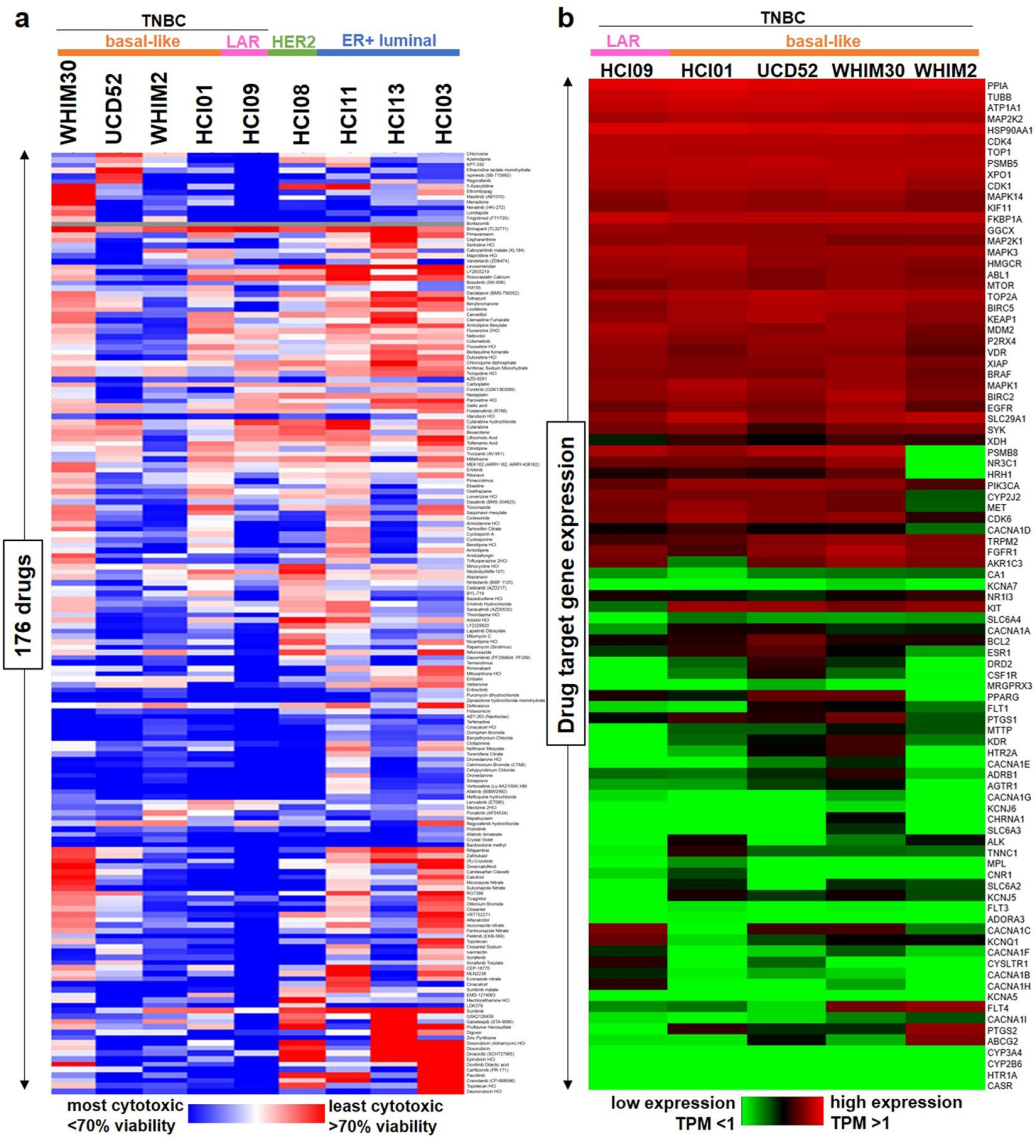
Selection of targeted drug candidates in TNBC PDXs based on a 1,363-drug screen. **(a)** Heatmap showing relative response to 176 drugs across PDXs of varying subtypes, selected based on efficacy in basal-like TNBC PDXs (HCI01, UCD52, WHIM2, WHIM30) on initial screening of 1,363 drugs at 10 µM. Hierarchical clustered cell viability data (average percent of vehicle) are represented in the heatmap for comparison of drug response across PDXs (n = 2 per PDX). The 1,363-drug screening data and 176-drug and target list are provided in Supplementary File S1. **(b)** Heatmap showing relative expression of target genes of the 176 selected drugs across TNBC PDXs. Clustered TPM values from PDX RNA-sequencing data (averaged for each PDX) are represented in the heatmap for analysis of target gene expression levels across PDXs.

Given the lack of successful targeted therapies currently available for the treatment of TNBC, and the superior clinical relevance of using PDX cultures as opposed to cell lines for assessing drug response in cancer^37^, we first sought to identify effective targeted agents through drug screening of breast cancer PDXs: basal-like TNBC (HCI01, HCI16, UCD52, WHIM2, WHIM30), luminal androgen receptor (LAR) subtype TNBC (HCI09), luminal ER-positive (HCI03, HCI11, HCI13), and HER2-enriched (HCI08). We characterized response profiles, in terms of percent cell viability, of these PDXs of varying breast cancer subtypes to 1,363 drugs, most of which are FDA-approved for various cancer/non-cancer indications (Supplementary File S1). This dataset is most appropriately useful for assessing drugs that are cytotoxic to tumor cells (less than 100% viability in response), as several drugs or classes of drugs, most notably histone deacetylase (HDAC) inhibitors, appeared to increase tumor cell viability, due to activation of the cytomegalovirus (CMV) promoter responsible for luciferase expression in the PDX models; HDACs are known to inactivate viral promoters^38^, and HDAC inhibitors have been shown to enhance CMV promoter activity^39–41^. It is possible that other drugs may affect CMV promoter activity as well. Using this drug screening dataset, we identified 176 drugs that were most cytotoxic across four of the basal-like PDXs (HCI01, UCD52, WHIM2, WHIM30) (Fig. 1a), encompassing an interestingly wide range of molecular targets, mechanisms of action, and indications (Supplementary File S1). The variety of proteins and pathways targeted by these drugs include the cell cycle, proteasome, ion channels, apoptosis pathways, calcium/vitamin D receptor signaling, EGFR and mitogen-activated protein kinase (MAPK) signaling, and serotonin signaling, as well as several non-human, microbial pathogen targets, indicating these drugs for treatment of a range of diseases, including cancer, cardiac arrhythmias, calcium imbalance, depression, and bacterial/viral/parasitic infections. Although several drugs of similar classes or with similar mechanisms of action (e.g. doxorubicin and epirucibin, fluoxetine and duloxetine, benidipine and amlodipine) clustered together in terms of PDX drug response profiles, most drugs of similar classes or mechanisms were part of distinct clusters.

Analysis of previous RNA-sequencing data^42^ revealed that about half of the genes encoding human targets of the 176 drugs are highly expressed in TNBC PDXs (Fig. 1b). Among the highly expressed genes in TNBC PDXs were cyclin-dependent kinase 4 (CDK4), proteasome subunit beta 5 (PSMB5), EGFR, BIRC5 (survivin), and vitamin D receptor (VDR), which encode the targets of abemaciclib (LY2835219), carfilzomib/bortezomib/ixazomib, afatinib, YM155, and calcitriol, respectively.

### Carfilzomib and afatinib have supra-additive trends when combined with other select targeted agents

**Figure 2:**
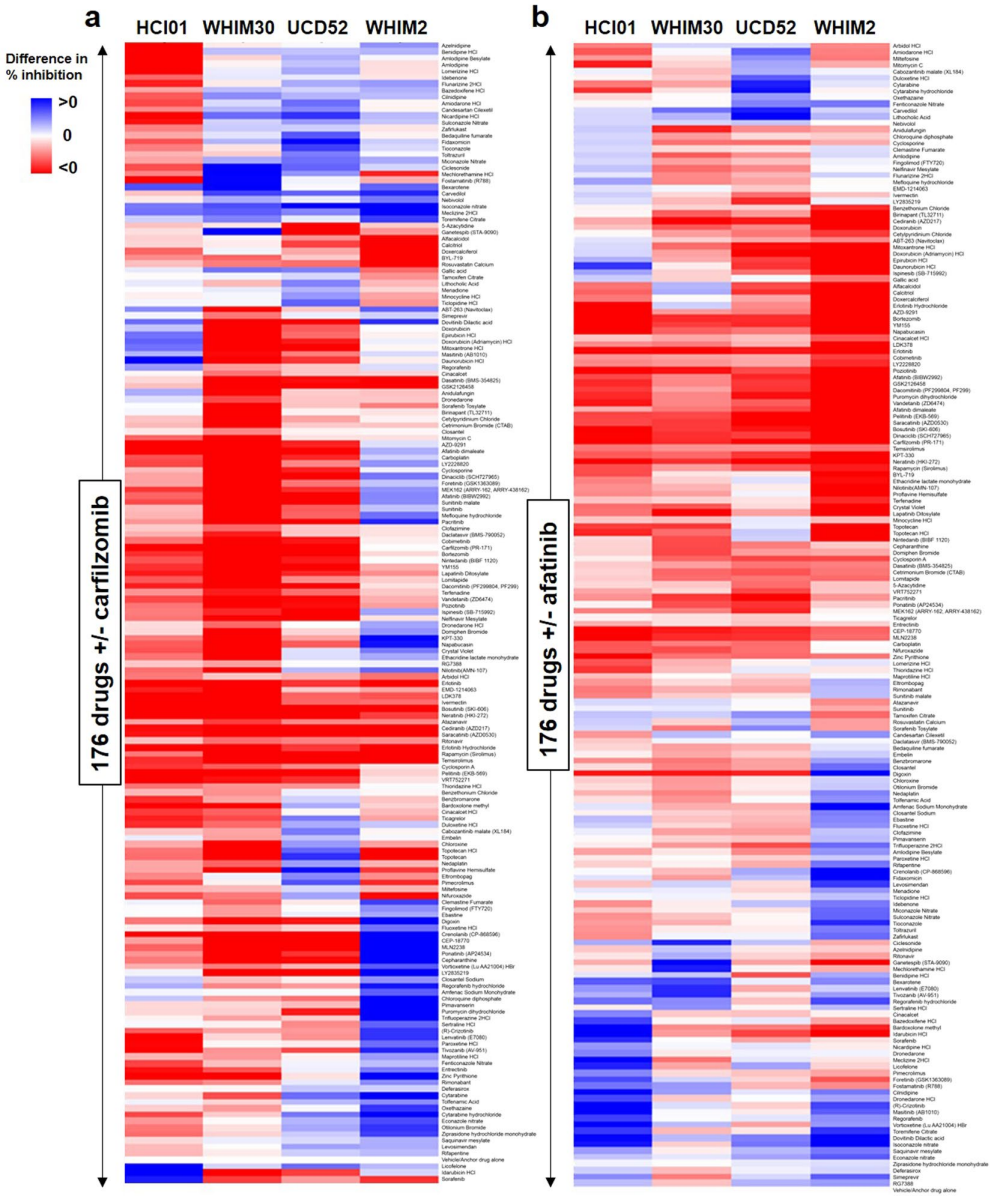
Efficacy of 176 selected drugs combined with carfilzomib or afatinib in basal-like TNBC PDXs. PDX cells (HCI01, UCD52, WHIM2, WHIM30) were treated with 176 drugs at 1 µM+/− carfilzomib or afatinib. Difference in percent inhibition of cell viability between each drug combination and each drug alone was calculated to assess for additive, supra-additive, or sub-additive trends: *(percent inhibition of combination) − [(percent inhibition of drug 1 alone)* + *(percent inhibition of drug 2 alone)]*. Heatmaps depict clustered differences in average percent inhibition between each of the 176 drugs combined with carfilzomib **(a)** or afatinib **(b)** compared with either drug alone; n = 2 for HCI01, UCS52, WHIM2; n = 3 for WHIM30. Differences in percent inhibition of 0 indicate additive trends (white), >0 indicate supra-additive trends (blue), and <0 indicate sub-additive trends (red). The 176 selected drugs and carfilzomib/afatinib combination data, along with confidence intervals and p-values, are provided in Supplementary File S2.

Although proteasome and EGFR inhibitors have demonstrated preclinical efficacy in TNBC, it is likely that synergistic combinations with other targeted agents are necessary to achieve efficacy that is sufficient for clinical success^43–49^. We therefore tested carfilzomib and afatinib in combination with each of the 176 selected drugs, at a 10-fold lower dose (1 µM) relative to prior screening assays, in four basal-like PDXs (HCI01, UCD52, WHIM2, WHIM30) (Supplementary File S2). Drug combination effects were assessed by calculating the difference in percent inhibition (efficacy) between each combination and each drug alone, with positive values indicating supra-additivity (efficacy of combination > sum of efficacies of each drug alone), zero indicating additivity (efficacy of combination = sum of efficacies of each drug alone), and negative values indicating sub-additivity (efficacy of combination < sum of efficacies of each drug alone). There was considerable heterogeneity in drug combination effects between the PDXs (Fig. 2), which is reflective of the heterogeneity in drug response seen in patients with the same tumor subtypes in the clinic. Given our goal of identifying treatments that have the potential to provide maximal clinical benefit for TNBC patients, we chose to focus on drugs that were effective across multiple PDX models of basal-like TNBC. Several drugs were found to have additive or supra-additive trends in at least two of the four basal-like PDXs when combined with carfilzomib (including benidipine, bexarotene, carvedilol, isoconazole, embelin, dronedarone, and abemaciclib) (Fig. 2a) or afatinib (including benidipine, bexarotene, carvedilol, isoconazole, fluoxetine, amiodarone, candesartan, and dovitinib) (Fig. 2b), providing several drugs/drug classes of interest for further studies. When mean differences in percent inhibition were analyzed across all four PDXs (relative to a difference in percent inhibition of zero), only isoconazole and meclizine were significantly supra-additive when combined with carfilzomib and only bexarotene was significantly supra-additive when combined with afatinib; all other drugs were either significantly sub-additive or not significant in either direction (Supplementary File S2).

### Carfilzomib, afatinib, and YM155 are cytotoxic to TNBC PDX cells

**Figure 3:**
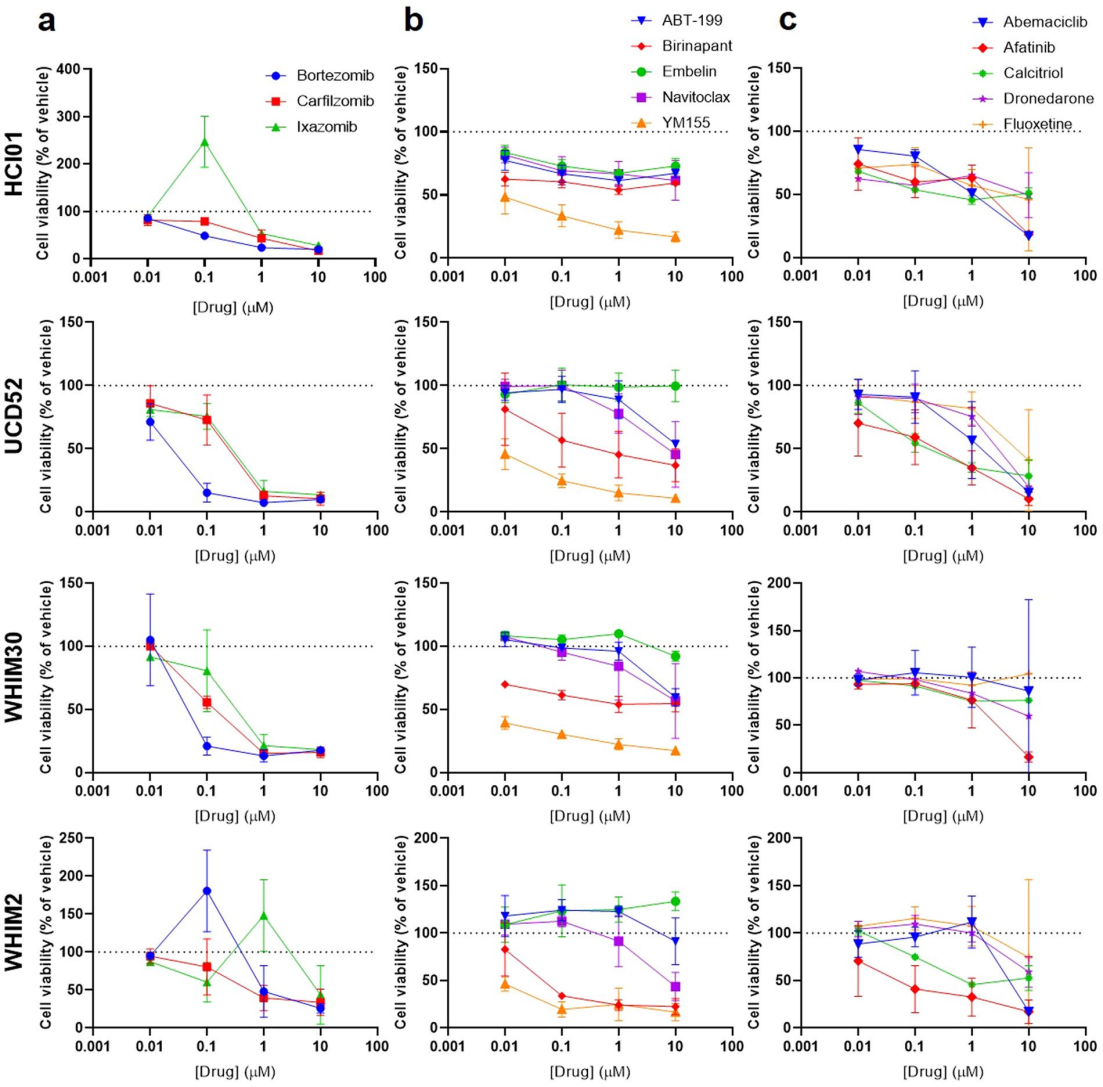
Dose responses of basal-like TNBC PDXs to selected classes of targeted therapeutics. Graphs depict cell viability (percent of vehicle) in response to increasing concentrations of the indicated drugs for each of four basal-like PDX lines (HCI01, UCD52, WHIM30, WHIM2): **(a)** proteasome inhibitors (carfilzomib, bortezomib, ixazomib); **(b)** drugs targeting apoptosis pathways (YM155, navitoclax, ABT-199, embelin, birinapant); and **(c)** EGFR inhibitor (afatinib), CDK4/6 inhibitor (abemaciclib), SSRI (fluoxetine), synthetic vitamin D3 (calcitriol), antiarrhythmic (dronedarone). Experiments were performed in triplicate. Error bars represent standard deviation between independent experiments. *p*-values are listed in Supplementary Table S1.

Given that the prior combination studies consisted of a single dose of each drug, this posed a significant limitation in that it was not possible to assess additive or supra-additive trends in combination with drugs that were highly cytotoxic as single agents at 1 µM, such as YM155 (Supplementary File S2). Therefore, several drugs/classes of drugs were selected for dose response testing to assess both potency and efficacy across the four basal-like PDX lines: carfilzomib, bortezomib, and ixazomib (proteasome inhibitors) (Fig. 3a); YM155 (survivin inhibitor), navitoclax and ABT-199 (B-cell lymphoma 2 (BCL2) inhibitors), embelin (X-linked inhibitor of apoptosis (XIAP) inhibitor), and birinapant (inhibitor of apoptosis (IAP) inhibitor/second mitochondria-derived activator of caspases (SMAC) mimetic), all of which promote apoptosis (Fig. 3b); afatinib (EGFR inhibitor), abemaciclib (CDK4/6 inhibitor), fluoxetine (selective serotonin reuptake inhibitor (SSRI)), calcitriol (synthetic vitamin D3), and dronedarone (ion channel blocker) (Fig. 3c). All p-values are listed in Supplementary Table S1. Proteasome inhibitors were significantly effective across the PDXs in the micromolar range; it should be noted that certain doses of ixazomib and/or bortezomib appeared to cause an increase in cell viability in HCI01 and WHIM2 at lower doses, followed by a decrease in viability with higher doses, which we believe to be due to proteasome inhibitor activity at CMV promoters causing an increase in expression of luciferase, as seen with HDAC inhibitors in the 1,363-drug screen. The survivin inhibitor YM155 was the most potent drug tested and was significantly effective across all four PDXs in the nanomolar range. Carfilzomib, YM155, and afatinib were selected for subsequent multiple-dose combination studies, given their efficacy across basal-like TNBC PDXs and the high expression of their drug targets in these tumor cells (Fig. 1b).

### Afatinib and YM155 are synergistically cytotoxic across TNBC PDXs

**Figure 4:**
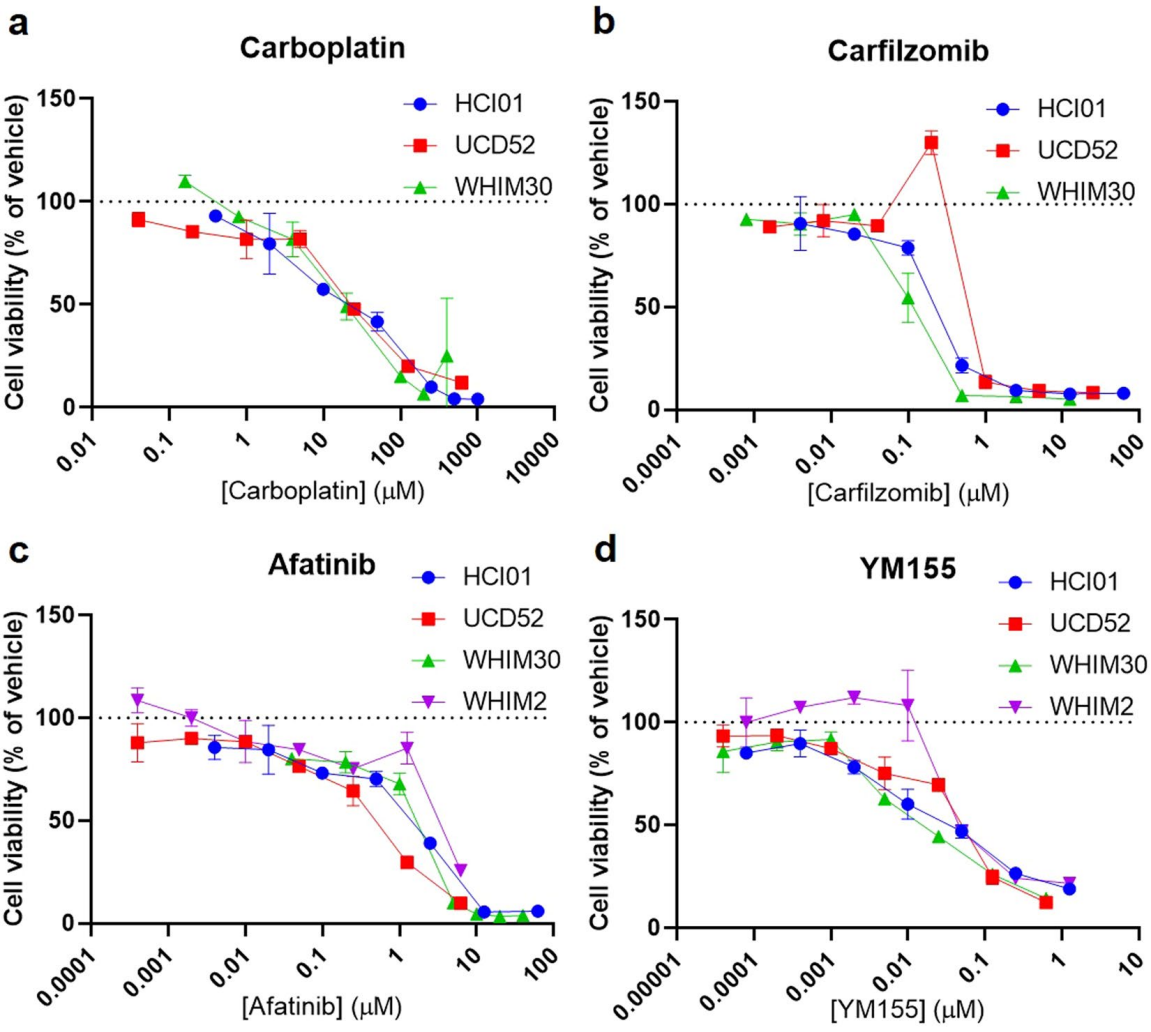
Dose responses of basal-like TNBC PDXs to four promising drug candidates. Graphs depict cell viability (percent of vehicle) in response to increasing concentrations of carboplatin **(a)** carfilzomib **(b)** afatinib **(c)** or YM155 **(d)** for each PDX line (HCI01, UCD52, WHIM30, WHIM2). Each experiment was performed in triplicate. Error bars represent standard deviation between independent experiments (n = 2 for each PDX); *p*-values are listed in Supplementary Table S3.

We next sought to identify synergistic combinations among drugs we have established as consistently effective with highly expressed drug targets in basal-like TNBC PDXs (carfilzomib, YM155, and afatinib), as well as carboplatin, a standard-of-care chemotherapeutic agent. HCI01, UCD52, and WHIM30 cells were treated with seven doses of each of these four drugs (WHIM2 cells with afatinib and YM155 only), and all possible two-drug combinations. Percent viability values were converted into fraction inhibition values (Fa). Drug doses were tailored for each PDX (Supplementary Table S2) based on prior dose response data to achieve a consistent dose response for each drug across the PDXs, and, as established previously, the four drugs were significantly cytotoxic to these PDX cells, and YM155 was the most potent of the four drugs tested in the PDXs (Fig. 4); all p-values are listed in Supplementary Table S3.

To identify synergistic drug combinations, data were analyzed using CompuSyn^50–52^ to determine combination index (CI) and dose-reduction index (DRI) values for each drug combination tested at a constant dose ratio. CI values indicate the effect of combining multiple drugs (synergistic, additive, or antagonistic); DRI values represent the fold decrease in the dose of a drug needed when in a combination to achieve the same efficacy (Fa) as the drug alone. Using this approach, drug combinations with CI values < 1 are synergistic, and DRI values > 1 are favorable given the concern for toxicity when combining multiple drugs. When assessing drug combinations for cancer treatment, these criteria are most important if met at high effect (Fa) levels, when the drug combinations are killing most of the tumor cells, as this is the goal of cancer therapy. We therefore considered any drug combination with CI < 1 and DRI > 1 (for both drugs in the combination) at Fa > 0.75 to be a promising combination.

**Figure 5:**
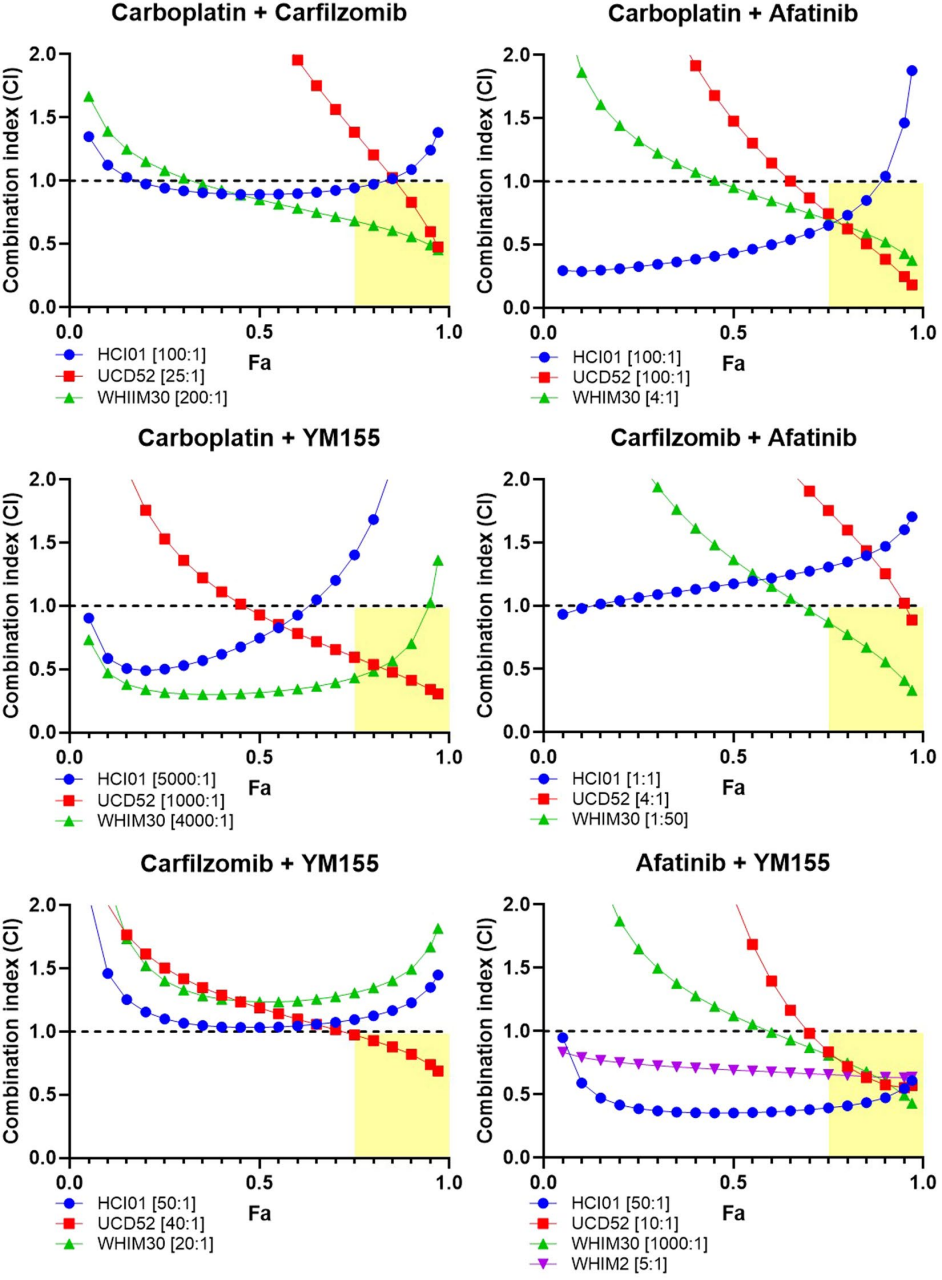
Drug combination analysis reveals synergism between afatinib and YM155 across four basal-like TNBC PDXs. PDX cells were treated with four drugs (carboplatin, carfilzomib, afatinib, and YM155), seven doses each, alone and in all possible two-drug combinations. Combination index (CI) values were generated using CompuSyn software, and Fa-CI plots were generated using constant dose ratio combination data for each of the six drug combinations in each of the PDXs. CI < 1 indicates synergism; CI = 1 indicates additivity; CI > 1 indicates antagonism. The regions highlighted in yellow are synergistic (CI < 1) at optimal effect levels (Fa > 0.75). Dose ratios (Drug1:Drug2) for each drug combination and PDX are indicated in the legend of each graph.

**Figure 6:**
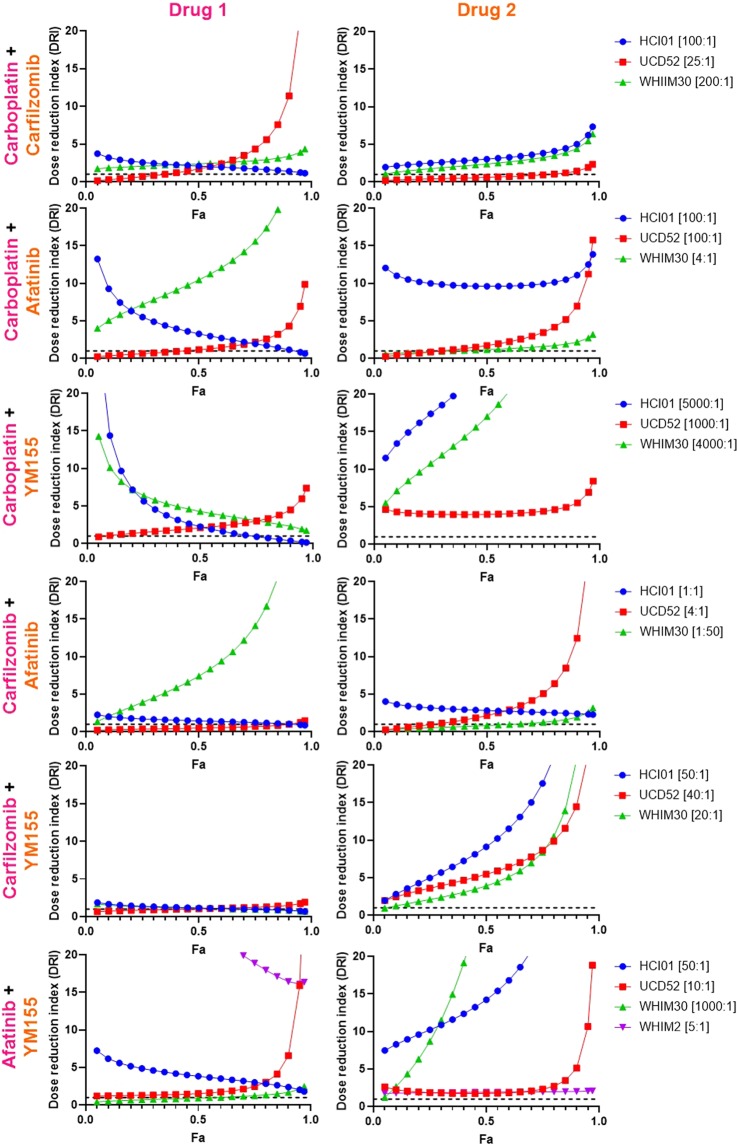
Drug combination analysis reveals favorable dose reduction of several drugs when combined with other agents in basal-like TNBC PDXs. PDX cells were treated with four drugs (carboplatin, carfilzomib, afatinib, and YM155), seven doses each, alone and in all possible two-drug combinations. Dose reduction index (DRI) values were generated using CompuSyn software, and Fa-DRI plots were generated using constant dose ratio combination data for each of the six drug combinations in each of the PDXs. DRI indicates the fold decrease in drug dose needed to achieve a given effect when in combination with another drug vs. as a single agent. DRI > 1 indicates favorable dose reduction; DRI < 1 indicates unfavorable dose reduction. Dose ratios (Drug1:Drug2) for each drug combination and PDX are indicated in the legend of each graph.

**Figure 7:**
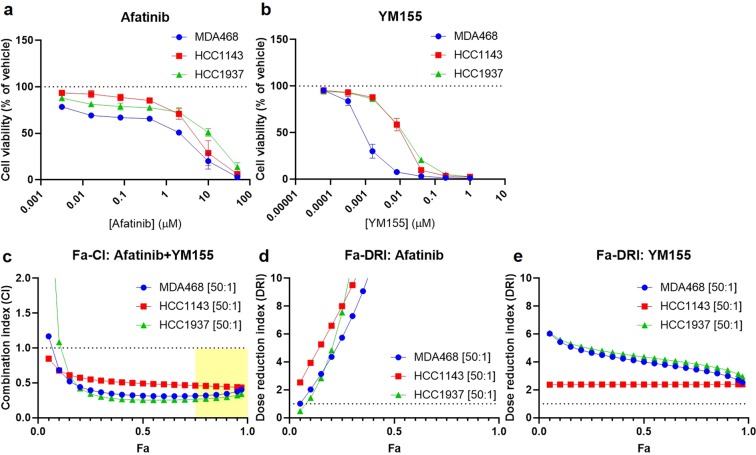
Afatinib and YM155 are synergistically cytotoxic to three basal-like TNBC cell lines. MDA468, HCC1143, and HCC1937 cells were treated in triplicate for 72 h *in vitro* with seven doses of afatinib or YM155, as well as all possible dose combinations of the two drugs. Graphs depict cell viability (percent of vehicle) of each of the three cell lines in response to afatinib **(a)** or YM155 **(b)**. Two independent experiments were performed for each cell line; error bars represent standard deviation; *p*-values are listed in Supplementary Table S4. Data were analyzed using CompuSyn software, and constant dose ratio combination data was used to generate a Fa-CI plot **(c)** and Fa-DRI plots for both afatinib **(d)** and YM155 **(e)**. Dose ratios (Drug1:Drug2) for each cell line are indicated in the graph legends. CI < 1 indicates synergism; CI = 1 indicates additivity; CI > 1 indicates antagonism. The region highlighted in yellow is synergistic (CI < 1) at optimal effect levels (Fa > 0.75). DRI indicates the fold decrease in drug dose needed to achieve a given effect when in combination with another drug vs. as a single agent. DRI > 1 indicates favorable dose reduction; DRI < 1 indicates unfavorable dose reduction.

Based on CI values: carboplatin was synergistic with carfilzomib, afatinib, and YM155 in UCD52 and WHIM30; carfilzomib was synergistic with afatinib in WHIM30 and with YM155 in UCD52; and afatinib was synergistic with YM155 in HCI01, UCD52, WHIM30, and WHIM2 (Fig. 5). DRI values were favorable for carboplatin when combined with carfilzomib, afatinib, or YM155 in UCD52 and WHIM30; for carfilzomib when combined with carboplatin, afatinib, or YM155 in HCI01, UCD52, and WHIM30; for afatinib when combined with carboplatin, carfilzomib, or YM155 in HCI01, UCD52, and WHIM30, and with YM155 in WHIM2; and for YM155 when combined with carboplatin, carfilzomib, or afatinib in HCI01, UCD52, and WHIM30, and with afatinib in WHIM2 (Fig. 6). Collectively, these data indicate that the combination of afatinib and YM155 is synergistic, with favorable dose reductions, across all four basal-like PDX lines tested. Afatinib and YM155 were also found to be effective as single agents (Fig. 7a,b) and synergistic (Fig. 7c) with favorable dose reductions (Fig. 7d,e) in three basal-like TNBC cell lines (MDA468, HCC1143, HCC1937), further confirming the efficacy and synergism of afatinib and YM155 in basal-like TNBC. All p-values for data shown in Fig. 7a,b are listed in Supplementary Table S4.

### Afatinib and YM155 reduce PDX mammary tumor growth *in vivo*

**Figure 8:**
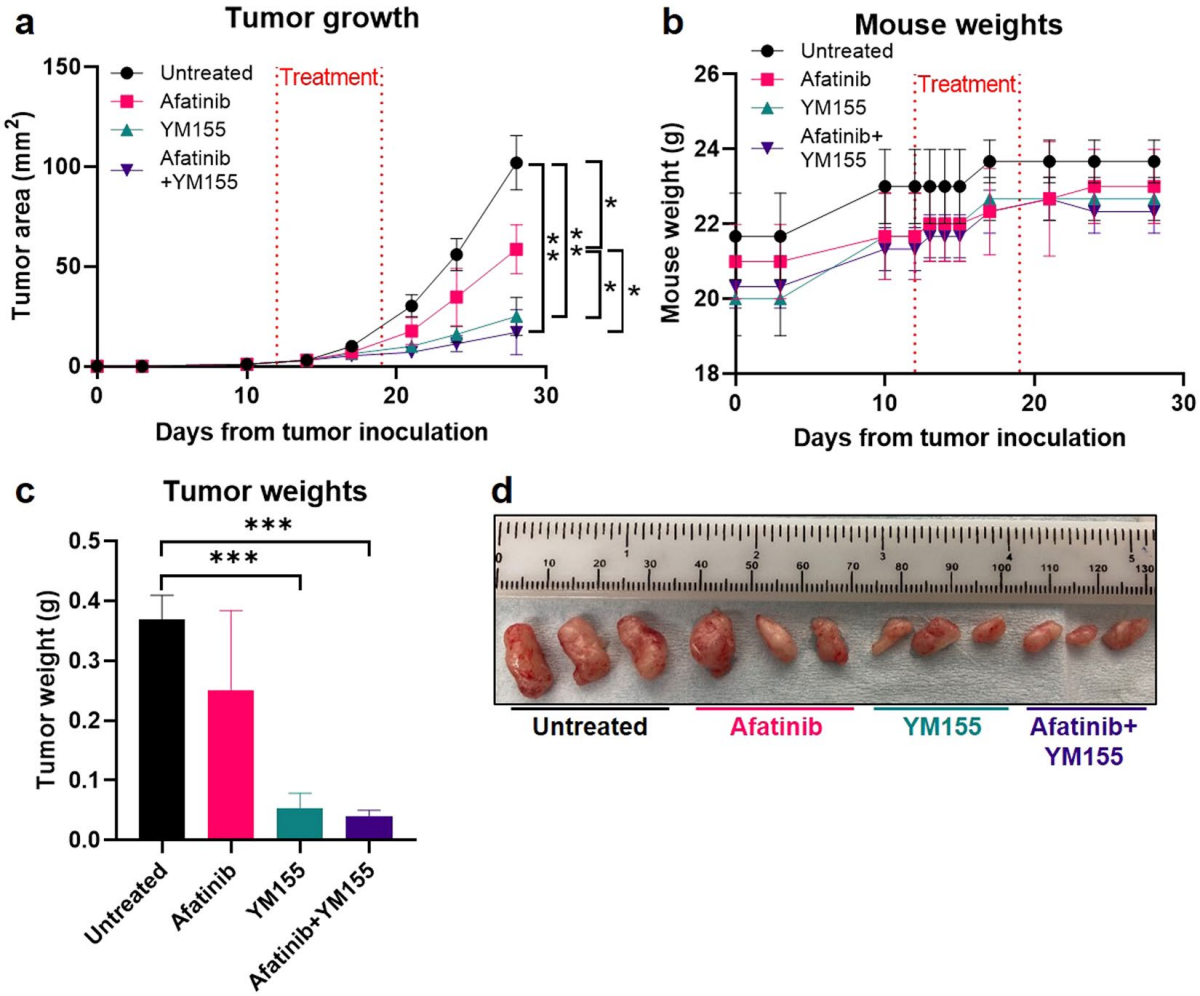
Afatinib and YM155 reduce PDX mammary tumor growth *in vivo*. HCI01 PDX cells were injected into the mammary fat pads of NSG mice. After 12 days of tumor growth, mice were divided into four groups (n = 3 mice per group): untreated, afatinib (25 mg/kg, daily oral gavage for 7 days), YM155 (5 mg/kg, 7-day continuous subcutaneous infusion via Alzet pump), and afatinib + YM155 (same doses and routes of administration as monotherapy groups). **(a)** Tumor area (length x width) over time for each treatment group, monitored via caliper measurements. The treatment period is indicated by red dotted lines. Error bars represent standard deviation. Significance is shown only for endpoint measurements (*p < 0.05, **p < 0.01); *p*-values for all timepoints are listed in Supplementary Table S5. **(b)** Mouse weights over time for each treatment group. The treatment period is indicated by red dotted lines. **(c)** Tumor weights for each treatment group, obtained after tumor removal at the study endpoint; ***p < 0.001; *p*-values are listed in Supplementary Table S5. **(d)** Photographs of mammary tumors for each treatment group at the study endpoint; ruler scale is mm.

To validate the efficacy of afatinib and YM155 *in vivo*, mice bearing HCI01 PDX mammary tumors were either untreated or treated with afatinib alone, YM155 alone, or afatinib + YM155. Both afatinib and YM155 as single agents, as well as afatinib and YM155 combined, significantly reduced mammary tumor growth over time compared to the control group (Fig. 8a). YM155 was significantly more effective as a single agent in reducing tumor growth compared to afatinib as a single agent, and the combination of afatinib + YM155 was significantly more effective than afatinib alone; there was no significant difference in tumor growth between YM155 alone and afatinib + YM155 treated groups (Fig. 8a). All p-values are listed in Supplementary Table S5. Importantly, mice did not display any signs of drug toxicity throughout or following the treatment period, and no considerable changes in mouse weight were observed in treated mice compared to control mice (Fig. 8b). Once tumors in control mice reached near protocol-defined burden, all mice were euthanized, and mammary tumors were removed. Mammary tumor weights were not significantly different between afatinib-treated and untreated mice, however tumor weights were significantly reduced in mice treated with YM155 as a single agent and combined with afatinib compared to control mice (Fig. 8c). All p-values are listed in Supplementary Table S5. Grossly, afatinib-treated tumors appeared slightly smaller, and YM155- and afatinib + YM155- treated tumors appeared considerably smaller, than control tumors (Fig. 8d).

### YM155 reduces EGFR expression in basal-like TNBC PDX cells

**Figure 9:**
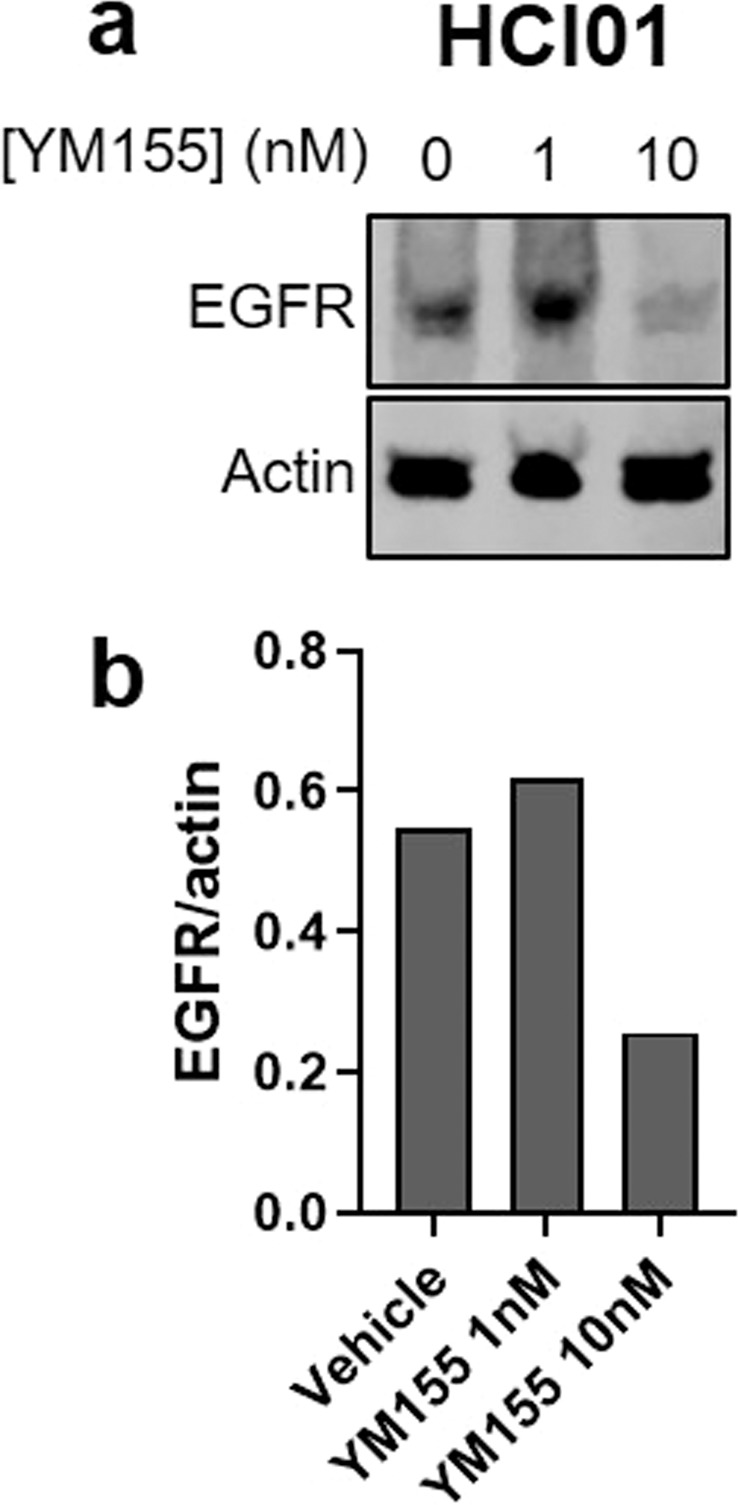
YM155 reduces EGFR expression in basal-like TNBC PDX cells. **(a)** Western blot showing EGFR expression in HCI01 cells treated with vehicle (DMSO) or YM155 (1 or 10 nM); actin was used as a loading control (100 µg per sample). Images are cropped blots showing proteins from different parts of the same gel. Corresponding full-length blots are shown in Supplementary Fig. S1. **(b)** Densitometry graph showing EGFR normalized to actin for each treatment condition. Samples were run on the same gel, and loading controls were run on the same blot.

To preliminarily explore potential crosstalk between the pathways targeted by afatinib and YM155, we performed Western blots to assess the effects of YM155 treatment on EGFR expression in the HCI01 PDX line. Interestingly, we found that YM155 treatment (at 10 nM) resulted in reduced EGFR protein expression in HCI01 cells compared to vehicle controls (Fig. 9a,b; Supplementary Fig. S1), indicating that YM155 has activity against the molecular target of afatinib in these basal-like TNBC cells.

### EGFR and BIRC5 are highly expressed in basal-like PDXs, cell lines, and patient tumors

**Figure 10:**
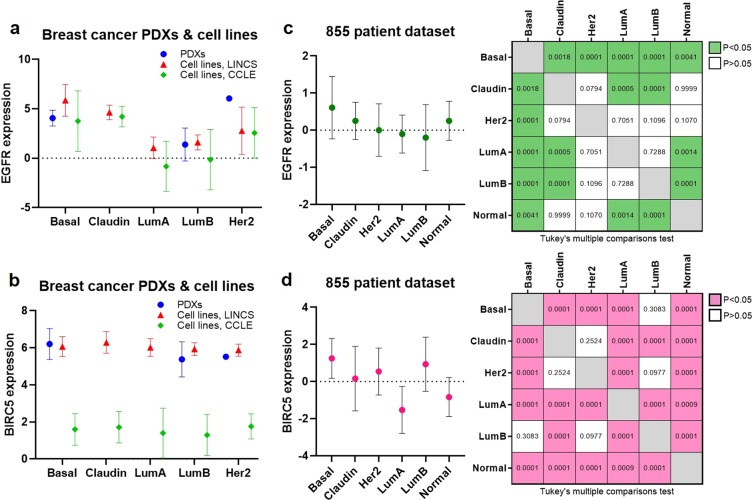
EGFR and BIRC5 are highly expressed in basal-like PDXs, cell lines, and patient tumors. RNA-sequencing data from breast cancer PDXs and cell lines (LINCS and CCLE databases) were used to assess expression levels of EGFR **(a)** and BIRC5 **(b)** according to intrinsic subtype: basal-like (Basal), claudin-low (Claudin), luminal A (LumA), luminal B (LumB), HER2-enriched (Her2). Gene expression data from 855 breast cancer patients were used to assess expression levels of EGFR **(c)** and BIRC5 **(d)** in patients according to intrinsic subtype: basal-like (Basal), claudin-low (Claudin), luminal A (LumA), luminal B (LumB), HER2-enriched (Her2), normal-like (Normal). Tukey’s multiple comparisons tests were used to analyze differences in expression levels of each gene between each subtype; tables in right panels depict *p*-values. PDX, cell line, and patient datasets were each grouped by breast cancer intrinsic subtype, and expression values for each gene were averaged; graphs depict the average (marker) and range (bars) of expression of EGFR or BIRC5 in each breast cancer subtype.

EGFR and BIRC5 (survivin) mRNA expression levels were assessed based on breast cancer intrinsic subtype using our PDX RNA-sequencing data, as well as RNA-sequencing data from two different breast cancer cell line gene expression databases: the Harvard Medical School (HMS) Library of Integrated Network-based Cellular Signatures (LINCS) Breast Cancer Profiling Project (http://lincs.hms.harvard.edu/db/datasets/20348/) and the Broad Institute Cancer Cell Line Encyclopedia (CCLE) (https://portals.broadinstitute.org/ccle). These analyses found that EGFR is most highly expressed in the basal-like subtype compared to the other subtypes in PDXs and cell lines (Fig. 10a), while BIRC5 expression is consistently high across all of the intrinsic subtypes in PDXs and cell lines (Fig. 10b). Analyses using an 855-patient breast cancer gene expression dataset^10,53,54^ identified that both EGFR and BIRC5 have significantly higher expression levels in basal-like patient tumors compared to those of other subtypes (Fig. 10c,d).

**Figure 11:**
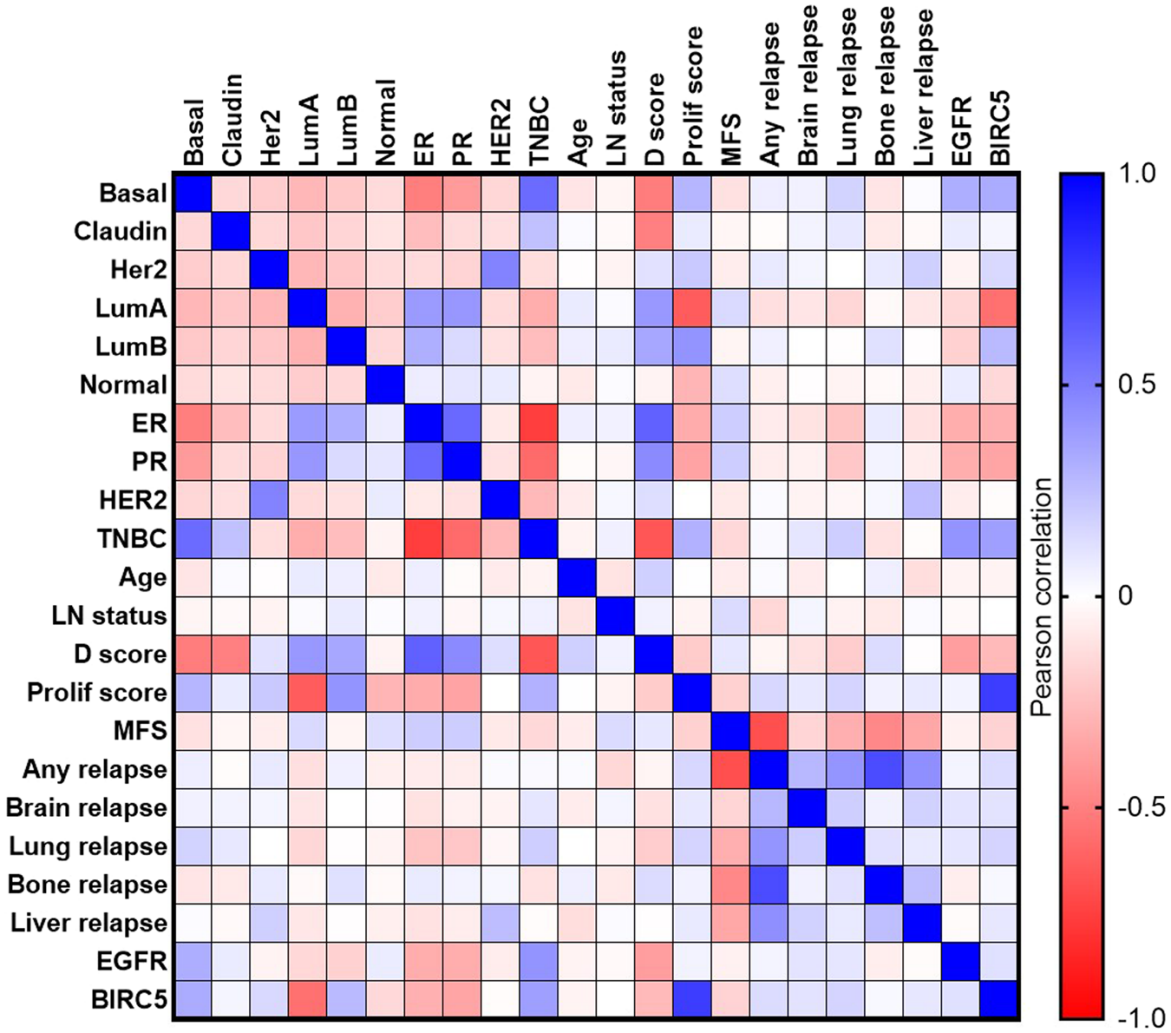
EGFR and BIRC5 expression correlate with clinical characteristics of patient tumors. Using an 855-patient dataset consisting of gene expression data as well as clinical information, Pearson correlations were performed to assess the relationships of EGFR and BIRC5 expression with clinical parameters. Intrinsic subtype: basal-like (Basal), claudin-low (Claudin), luminal A (LumA), luminal B (LumB), HER2-enriched (Her2), normal-like (Normal). Receptor status: estrogen receptor (ER), progesterone receptor (PR), human epidermal growth factor receptor 2 (HER2), triple-negative breast cancer (TNBC). Other parameters: patient age, lymph node (LN) status, differentiation (D) score, proliferation (Prolif) score. Clinical outcomes: metastasis-free survival (MFS), relapse-free survival (any relapse, brain, liver, lung, bone). Heatmap depicts Pearson correlation values for each comparison of parameters: negative correlation (red), no correlation (white), positive correlation (blue).

Pearson correlation analysis was performed to assess the relationships between EGFR and BIRC5 expression and clinical characteristics of breast cancer patients using the 855-patient dataset. This revealed positive correlations of both EGFR and BIRC5 expression with basal-like triple-negative tumors, and negative correlations of both EGFR and BIRC5 expression with luminal ER/PR-positive tumors and differentiation score (Fig. 11). In addition, BIRC5 expression showed a strong positive correlation with proliferation score (Fig. 11).

### EGFR and BIRC5 expression are negatively associated with patient outcomes

**Figure 12:**
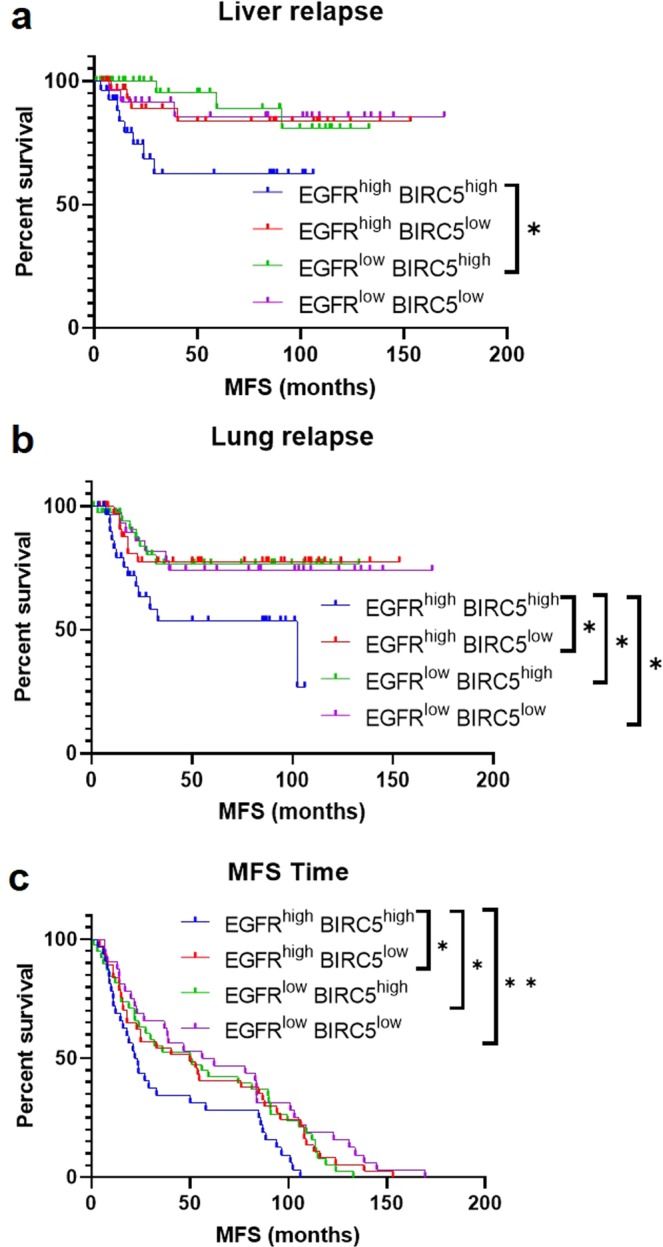
EGFR and BIRC5 expression are negatively associated with metastasis-free survival in patients with basal-like tumors. Using an 855-patient breast cancer dataset, patients with basal-like tumors (N = 140) were divided into four groups based on expression levels of EGFR and BIRC5: EGFR^high^BIRC5^high^, EGFR^high^BIRC5^low^, EGFR^low^BIRC5^high^, EGFR^low^BIRC5^low^. Kaplan-Meier curves were generated to assess the effect of high versus low expression of the two genes on **(a)** liver relapse-free survival, **(b)** lung relapse-free survival, and **(c)** metastasis-free survival (MFS) time. Log-rank tests were performed to determine statistical significance (*p < 0.05, **p < 0.01); *p*-values are listed in Supplementary Table S6.

The 855-patient dataset was used to determine the effect of EGFR and BIRC5 expression levels on clinical outcomes for patients with basal-like tumors (N = 140) in terms of metastasis-free survival (MFS) time, as well as relapse-free survival pertaining to liver and lung metastases. Basal-like patients were divided into four groups based on EGFR/BIRC5 expression levels: EGFR^high^BIRC5^high^, EGFR^high^BIRC5^low^, EGFR^low^BIRC5^high^, EGFR^low^BIRC5^low^. Kaplan-Meier analyses revealed that patients with EGFR^high^BIRC5^high^ tumors had significantly reduced liver relapse-free survival relative to those with EGFR^low^BIRC5^high^ tumors (Fig. 12a) as well as significantly reduced lung relapse-free survival compared to those with EGFR^high^BIRC5^low^, EGFR^low^BIRC5^high^, and EGFR^low^BIRC5^low^ tumors (Fig. 12b). Patients with EGFR^high^BIRC5^high^ tumors also had significantly shorter MFS times compared to those with EGFR^high^BIRC5^low^, EGFR^low^BIRC5^high^, and EGFR^low^BIRC5^low^ tumors (Fig. 12c). All p-values are listed in Supplementary Table S6. Collectively, these results indicate that high tumor expression levels of both EGFR and BIRC5 are associated with more rapid development of liver and lung metastases in patients compared to tumors with low expression of one or both of these genes.

## Discussion

Despite decades of translational research, no targeted therapeutics have yet been FDA-approved for the treatment of TNBC. Although several classes of targeted drugs have shown promise in preclinical studies, most of these drugs have failed in clinical trials, and it is likely that effective synergistic combination regimens are needed to successfully combat this disease^11^. One factor that certainly can contribute to the discrepancy in results between preclinical studies and clinical trials is the use of immortal cell line models for preclinical drug response testing. Cell lines have been shown to undergo considerable changes while in culture that can affect drug response, whereas three-dimensional PDX cultures have been shown to more faithfully maintain tumor cell morphology, gene expression, and drug response profiles^55–57^. Indeed, we have previously found that two of the breast cancer PDXs employed in the present studies maintain the gene expression profiles of their *in vivo* counterparts after seven days in cell culture^36^. Therefore, preclinical drug screening studies using PDXs are more likely than those using cell lines to be indicative of *in vivo* efficacy, and *in vivo* PDX drug studies are more likely to predict clinical potential^37^. Thus, the studies presented herein employ breast cancer PDX models, in addition to cell lines, to assess drug response.

Through screening of 1,363 drugs in ten PDXs of varying breast cancer subtypes, we have generated a drug response dataset that can be used to assess and compare drug response profiles of patient-derived breast cancer cells to many specific drugs or classes of drugs, currently approved for a wide range of clinical indications. From this dataset, we identified 176 drugs that were most effective in four basal-like TNBC PDXs, and through a series of subsequent drug screening assays, we selected four drugs of interest for combination testing and Chou-Talalay analysis: carfilzomib (proteasome inhibitor), afatinib (EGFR inhibitor), YM155 (survivin inhibitor), and carboplatin (standard-of-care chemotherapeutic). In addition to their efficacy in screening experiments, the former three drugs were of interest given the high level of expression of the genes encoding their targets. Of the six two-drug combinations, only afatinib and YM155 were found to be synergistic in the four basal-like PDXs tested, as well as in three cell lines. Notably, this combination was also favorable given the reduced dose of each drug required to achieve a given level of efficacy when combined with one another, suggesting that this drug combination could potentially minimize toxicity associated with combining multiple drugs. Indeed, our *in vivo* study employing the HCI01 PDX model not only demonstrated that both drugs, as single agents and in combination, were efficacious in reducing mammary tumor growth, but also that both drugs were very well-tolerated, with no observable signs of toxicity.

It should be noted that, although the *in vivo* study showed a greater reduction in HCI01 tumor growth when afatinib was combined with YM155 compared to afatinib treatment alone, there was no significant difference in tumor growth between mice treated with the combination versus YM155 alone—both YM155-containing treatment groups showed a marked reduction in tumor growth, regardless of the presence or absence of afatinib. This indicates that the efficacy of the drug combination was dominated by the effects of YM155, therefore synergism was not discernible. Nevertheless, the efficacy of both afatinib and YM155 *in vivo*, and their minimal toxicity profiles, further suggest that these drugs are promising candidates for TNBC treatment.

YM155 is an investigational inhibitor of survivin expression that has shown promise in preclinical models of TNBC^58,59^, drug-resistant ER-positive breast cancer^60,61^, and other solid tumor types^62–67^. BIRC5, the gene encoding survivin, is upregulated in many human cancers^68^, and in the breast cancer PDXs and cell lines employed in our study, and has been shown to have low levels of expression in normal tissue types^69,70^, which makes survivin an appealing drug target. The preclinical success of YM155 has led to its testing in several clinical trials, one of which was focused on combining YM155 with docetaxel in HER2-negative breast cancer (including TNBC), however this trial did not find significant benefit of the combination relative to docetaxel as a single agent^71^. The failure of preclinical drug studies to translate into clinical success is not uncommon. Thus, although YM155 was highly effective as a single agent in reducing mammary tumor growth in the *in vivo* study presented herein, its clinical track record to date suggests that its preclinical monotherapeutic efficacy would be unlikely to translate into success in clinical trials, whether or not it is combined with standard-of-care chemotherapeutics. It is likely that identification of synergistic combinations incorporating YM155 would maximize its efficacy and potential for clinical benefit.

Our present findings indicate that, in several basal-like TNBC PDX models, YM155 is synergistic with afatinib, an EGFR inhibitor currently approved for the treatment of non-small cell lung cancer. EGFR is expressed in a large percentage of TNBCs^16,72^, including TNBC PDX models and the cell lines used in this study, and it has been explored as a potential therapeutic target in this disease^11,73,74^. However, EGFR inhibitors and anti-EGFR antibodies have thus far been unsuccessful in TNBC clinical trials^75–80^, suggesting that, like for YM155, more effective combinations must be identified to maximize its efficacy. Based on our collective findings, we propose that YM155 and afatinib could potentially enhance each other’s efficacy in TNBC. Interestingly, YM155 has been shown to reduce EGFR expression and tumor cell proliferation and survival in pancreatic cancer^81^ as well as EGFR-positive non-small cell lung cancer, in which YM155 was found to be synergistic with afatinib^82^ and other EGFR inhibitors^83^, to reverse resistance to the EGFR inhibitor erlotinib in EGFR-mutant lung cancer^84^, and to inhibit EGFR autophosphorylation which promotes lung cancer stemness^85^. The EGFR inhibitor lapatinib was also found to enhance the efficacy of YM155 in neuroblastoma by inhibiting drug efflux through the ABCB1 transporter^86^.

Mechanisms of synergism between YM155 and EGFR inhibitors such as afatinib have not yet been extensively explored in breast cancer. We have demonstrated herein that YM155 reduces EGFR expression in a PDX model of basal-like TNBC, consistent with the aforementioned studies in pancreatic and lung cancer. Based on this finding, and on the aforementioned studies in other cancer types, we can postulate that YM155, in addition to promoting tumor cell apoptosis by inhibiting survivin, downregulates EGFR expression in breast cancer cells that highly express both BIRC5 and EGFR. When YM155 and afatinib are combined, this may potentiate the inhibition of EGFR-mediated pathways, leading to enhanced inhibition of tumor cell proliferation and survival. In turn, afatinib may enhance the efficacy of YM155 in breast cancer cells by inhibiting YM155 efflux; indeed, afatinib has been shown to inhibit the ABCB1 drug efflux transporter in ovarian cancer^87^ and the ABCG2 drug efflux transporter in other cancer types^88^. Further investigation of the mechanisms underlying the synergism between afatinib and YM155 in basal-like TNBC is warranted to explore these and alternative possibilities.

Our studies collectively provide compelling evidence that the combination of afatinib and YM155 holds promise for potential clinical benefit in the treatment of basal-like TNBC. This is further supported by our analyses of the 855-patient gene expression and clinical dataset. Both EGFR and BIRC5 were found to have the highest expression levels in basal-like tumors compared to other subtypes. In addition, both genes were positively correlated with basal-like triple-negative tumor status, and negatively correlated with luminal ER/PR-positive tumor status and differentiation score. BIRC5 was also positively correlated with proliferation score, as this is one of the 11 proliferation markers that is included in the PAM50 gene list, which is used clinically for breast cancer subtyping and predicting patient prognosis^89^. Taken together with the aforementioned studies demonstrating the prevalence and functions of these genes in cancer, these findings suggest that both EGFR and BIRC5 may be important drug targets in basal-like TNBC. Our analyses of the 855-patient dataset further revealed that high co-expression of EGFR and BIRC5 was associated with significantly reduced metastasis-free survival time and relapse-free survival specifically in the liver and lung. This suggests that co-targeting of EGFR and BIRC5 may have significant clinical impacts for patients with advanced basal-like TNBC, who currently face considerable limitations in treatment options and bleak outcomes relative to patients with tumors of other, currently targetable, subtypes. Based on our collective findings, the combination of afatinib and YM155, and combinations incorporating other EGFR and survivin inhibitors, warrant further investigation as novel therapeutic regimens for the treatment of basal-like TNBC.

In conclusion, the studies reported herein provide a valuable 1,363-drug response dataset, employing clinically relevant PDX models, that has the potential to inspire and inform many future studies focusing on therapeutic development in breast cancer. Using this dataset to inform more focused follow-up screening studies, with an emphasis on basal-like TNBC, we identified a promising synergistic drug combination that, to our knowledge, has not yet been examined in the context of this disease. Based on our collective findings and on previous research in other cancers, we believe that, upon further preclinical investigation, the combination of afatinib and YM155, and perhaps other EGFR and survivin inhibitors, could potentially be incorporated into novel therapeutic regimens for eventual clinical testing in humans. Furthermore, additional therapeutic strategies that may be explored based on our drug screening dataset, such as the repurposing of non-cancer therapeutics for breast cancer treatment, have the potential to make major translational impacts on treatment decisions, clinical outcomes, and quality of life for patients with advanced breast cancer.

## Methods

### Breast cancer PDX models and preparation of tumor cell suspensions

Breast cancer PDX models of varying subtypes were used in these studies: triple-negative, basal-like (HCI01, HCI16, UCD52, WHIM2, WHIM30); triple-negative, luminal androgen receptor (LAR) type (HCI09); ER-positive, luminal (HCI03, HCI11, HCI13); and HER2-enriched (HCI08). HCI01, HCI03, HCI08, HCI09, HCI11, HCI13, and HCI16 were obtained from the Huntsman Cancer Institute, University of Utah; WHIM2 and WHIM30 were obtained from Washington University, St. Louis; UCD52 was obtained from the University of Colorado. All studies involving mice were approved by the Virginia Commonwealth University (VCU) Institutional Animal Care and Use Committee (IACUC) (Protocol# AD10001247; approved June 29, 2018), and all experiments were performed in accordance with IACUC guidelines and regulations. Tumor fragments were grown in the fourth mammary fat pads of female non-obese diabetic severe combined immunodeficient gamma (NSG) mice. Established tumors were removed from mice, finely chopped, and digested for 1 h at 37 °C in DMEM/F12 containing 5% fetal bovine serum (FBS), 300 U/ml collagenase (Sigma), and 100 U/ml hyaluronidase (Sigma). Digested tumor tissue was then resuspended in ammonium chloride and trypsinized to generate single cell suspensions. Tumor cells were transduced with a lentivirus (BLIV101PA-1, Systems Biosciences) encoding green fluorescent protein (GFP) and luciferase (Luc), and GFP-Luc expressing tumor cells were suspended 1:1 in Matrigel (Corning) and injected into the fourth mammary fat pads of NSG mice (500,000 cells per injection). Mammary tumors were removed for experimental use once they reached approximately 100mm^2^ by caliper measurement. Tumors were processed into single cell suspensions as described above.

### Breast cancer cell lines

Three basal-like TNBC cell lines, MDA468, HCC1143, and HCC1937, were employed to validate the results of PDX studies. MDA468 cells were provided by Dr. Youngman Oh (VCU Department of Pathology); HCC1143 and HCC1937 cells were obtained from the American Type Culture Collection (ATCC). Cell lines were cultured in RPMI-1640 GlutaMAX medium (ThermoFisher Scientific) supplemented with 10% FBS and penicillin/streptomycin.

### Cell viability assays

For PDX cell viability assays, PDX cells were plated in 96-well plates at 25,000 cells per well in M87 medium^33^ and treated with drugs for 72 h, followed by imaging and measurement of luciferase activity (total photon flux per second) two minutes after the addition of D-luciferin (15 mg/ml; GoldBio) to each well (1/10 of total volume per well), using the IVIS Spectrum *In Vivo* Imaging System (Xenogen IVIS-200) and living image software (PerkinElmer), as described in our previous work^36^. For cell line viability assays, MDA468, HCC1143, or HCC1937 cells were plated in 96-well plates at 5,000 cells per well in complete RPMI-1640 GlutaMAX medium, cultured overnight to allow for adherence, and subsequently treated with drugs for 72 h. Viability of cell lines was measured using the CellTiter-Glo Luminescent Viability Assay (Promega), according to the provided protocol.

### *In vitro* drug screening studies

PDX tumor cells (HCI01, HCI16, UCD52, WHIM2, WHIM30, HCI08, HCI09, HCI03, HCI11, HCI13) were treated with 1,363 drugs (ApexBio DiscoveryProbe FDA-approved Drug Library), at 10 µM per drug, and cell viability was measured after 72 h as described above. Drug response was assessed and compared between drugs and PDXs by calculating the percent of vehicle (0.1% DMSO) viability for each drug-treated well. Replicates were then averaged for each PDX and analyzed based on breast cancer subtype, with a focus on identifying promising targeted therapeutic candidates for basal-like TNBC using four PDXs of this subtype (HCI01, UCD52, WHIM2, WHIM30). To select drug candidates for further studies, drug response data for each of these four PDXs were ranked in order of increasing efficacy (decreasing % of vehicle viability), and the 200 most effective drugs were chosen for each individual PDX line. We then used a Venn diagram (https://bioinfogp.cnb.csic.es/tools/venny/) to determine the extent of overlap in the most effective drugs across the four PDXs. Based on this analysis, we selected 176 drugs for further testing in basal-like TNBC models, consisting of: 1) 71 drugs that overlapped across all four PDXs, 2) 53 drugs that overlapped in three of the PDXs, 3) 48 drugs that overlapped in two of the PDXs, 4) two drugs that were exclusive to one of the PDXs (erlotinib and carboplatin), and 5) two drugs that were not included on these lists but were of interest from a mechanistic standpoint, to compare with other drugs with similar mechanisms of action (birinapant and bortezomib). All subsequent drug studies were performed using the same drug stock solutions purchased from ApexBio.

### Single-dose drug combination studies

All drug combination studies were carried out *in vitro* using the same cell viability assay methods described above. For initial combination studies, the 176 selected drugs were tested on PDX cells (HCI01, UCD52, WHIM2, WHIM30) at 1 µM alone and in combination with the proteasome inhibitor carfilzomib (10 nM for HCI01, UCD52, and WHIM30; 100 nM for WHIM2) or the EGFR inhibitor afatinib (10 nM for HCI01, UCD52, and WHIM2; 1 µM for WHIM30). To assess for additive/supra-additive/sub-additive trends (defined here based on whether the efficacy of a combination was equal to/greater than/less than the sum of the efficacies of each drug alone), percent cell viability values were used to calculate the difference in percent inhibition between each drug as a single agent and in combination: (percent inhibition of combination) − [(percent inhibition of drug 1 alone) + (percent inhibition of drug 2 alone)]. Using this approach, if the calculated value for a combination is greater than one, the combination has supra-additive trends; if it is zero, it has additive trends; if it is less than one, it has sub-additive trends. The data generated in these studies were used to help select drugs of interest for more expansive combination testing, described below.

### Multiple-dose drug combination studies

Based on initial screening and single-dose drug combination data, as well as drug target gene expression data, 13 drugs were selected for dose response analysis: three proteasome inhibitors (carfilzomib, bortezomib, ixazomib), five drugs that target apoptosis pathways (YM155, navitoclax, ABT-199, embelin, birinapant), an EGFR inhibitor (afatinib), a CDK4/6 inhibitor (abemaciclib), a SSRI (fluoxetine), synthetic vitamin D3 (calcitriol), and an antiarrhythmic agent (dronedarone). These drugs included the two drugs tested in combination with the 176 drugs in the single-dose combination screen (carfilzomib and afatinib), one of the most effective of the 176 drugs in the prior screening studies (YM155), drugs with similar mechanisms of action (two additional proteasome inhibitors and four additional drugs that target apoptosis pathways), and drugs with mechanisms that are not typically targeted in cancer therapy (calcitriol, dronedarone, and fluoxetine). Basal-like TNBC PDX cells (HCI01, UCD52, WHIM2, WHIM30) were treated with increasing concentrations of each of the 13 drugs (ranging from 0.1–10 µM) for 72 h, followed by cell viability measurement. Based on potency and efficacy across the four PDXs, three of these drugs (carfilzomib, YM155, and afatinib) were selected for subsequent combination testing, along with carboplatin, a chemotherapeutic agent that is part of the standard-of-care regimen for TNBC and that we have previously tested in several PDXs^36^. Pharmaceutical-grade carboplatin was obtained from the VCU Dalton Oncology Clinic. PDX cells (HCI01, UCD52, WHIM30) were treated for 72 h *in vitro* with 7 doses of each drug alone, and with all possible two-drug combinations: carboplatin + carfilzomib, carboplatin + afatinib, carboplatin + YM155, carfilzomib + afatinib, carfilzomib + YM155, afatinib + YM155. Afatinib + YM155 was additionally tested in the WHIM2 PDX model, as well as three breast cancer cell lines (MDA468, HCC1143, HCC1937). Two independent experiments, each in triplicate, were performed for each PDX/cell line. Fa values were calculated using percent viability values for each drug and drug combination. Triplicate Fa values were averaged, and data were analyzed for drug combination effects using the CompuSyn software, which employs the Chou-Talalay method^50–52^. CI and DRI values, generated by CompuSyn software simulation, were averaged for each PDX/cell line and used to generate Fa-CI and Fa-DRI plots for each constant-ratio drug combination.

### Data clustering

Data were hierarchically clustered using Cluster 3.0, and heatmaps were generated using Java Treeview. This was performed for drug response data (percent cell viability in response to the 176 drugs selected from initial screening, and difference in percent inhibition of the 176 drugs alone and in combination with carfilzomib or afatinib), as well as gene expression data (transcripts per million (TPM) values) to assess drug target expression across the PDXs. The latter data were obtained from previous RNA-sequencing of PDXs^42^, and are publicly available in the NCBI Gene Expression Omnibus (GEO Accession: GSE118942). Data were clustered by both drugs/genes and PDX line.

### *In vivo* PDX drug treatment studies

HCI01 cell suspensions were prepared from mammary tumors as described above. After 12 days of tumor growth, monitored by weekly caliper measurements, mice were divided into four treatment groups: untreated (n = 3), afatinib (n = 3), YM155 (n = 3), and afatinib + YM155 (n = 3). Afatinib (AChemBlock) was dissolved in 1% methylcellulose + 0.1% Tween-80 and administered at 25 mg/kg via daily oral gavage for 7 days. YM155 (Adooq Bioscience) was dissolved in saline and administered at 5 mg/kg as a 7-day continuous subcutaneous infusion via Alzet pump (Alzet 1007D). Alzet pumps were implanted subcutaneously on the back, posterior to the scapulae. During and following the treatment period, tumor growth was monitored via biweekly caliper measurements. Mice were weighed and observed regularly throughout the study for signs of illness or distress related to tumor growth and/or drug toxicity. All mice were euthanized by CO_2_ asphyxiation followed by cervical dislocation once tumors of untreated mice reached near protocol-defined tumor size limits. Tumors were then immediately removed, weighed *ex vivo*, and photographed.

### Western blot studies

HCI01 PDX cell suspensions were prepared from mammary tumors as described above, plated in 100 mm dishes at 5 million cells per dish in M87 medium, and treated for 24 h with vehicle (DMSO) or YM155 (1 or 10 nM). For protein extraction, treated HCI01 cell suspensions were pelleted and resuspended in Pierce RIPA buffer (ThermoFisher Scientific, 89900) + protease inhibitor (ThermoFisher Scientific, A32963) for cell lysis, and centrifuged at max speed at 4 °C for 15 min to collect protein lysates. Protein concentrations were determined using Pierce BCA Protein Assay Kit (ThermoFisher Scientific, 23225). Proteins were resolved by SDS-PAGE and transferred to Immobilon-FL membranes (Millipore), which were then blocked in Odyssey Blocking Buffer in TBS (Li-Cor) for 1 h at room temperature. Primary and secondary antibodies were diluted in Odyssey Blocking Buffer in TBS (Li-Cor) + 0.1% Tween-20. Membranes were incubated for 1 h at room temperature with rabbit anti-β-actin (1:1000; 13E5, CST #4970) and overnight at 4 °C with rabbit anti-EGFR (1:1000, D38B1, CST #4267). For detection, membranes were incubated with IRDye 680RD donkey anti-rabbit secondary antibody (1:10,000; Li-Cor, 926-68073) for 1 h at room temperature. All washes were performed using TBS-T. Membranes were imaged using the Odyssey Fc Imaging System (Li-Cor). Densitometry analysis was performed using ImageJ software; EGFR was normalized to actin.

### Analysis of EGFR and BIRC5 gene expression in PDXs, cell lines, and patients

Expression levels of EGFR and BIRC5 were assessed using a PDX RNA-sequencing dataset^42^, as well as RNA sequencing data from two publicly available breast cancer cell line gene expression databases: the Harvard Medical School (HMS) Library of Integrated Network-based Cellular Signatures (LINCS) Breast Cancer Profiling Project (http://lincs.hms.harvard.edu/db/datasets/20348/) and the Broad Institute Cancer Cell Line Encyclopedia (CCLE) (https://portals.broadinstitute.org/ccle). Expression of the two genes was also assessed using a breast cancer patient dataset consisting of microarray gene expression data and clinical data^53,54^ from 855 patients; this dataset was generated by combining four breast cancer microarray datasets (GSE2034, GSE12276, GSE2603, and NKI295)^10^. PDXs, cell lines (from each database separately), and the 855-patient data were each grouped based on breast cancer intrinsic subtype, and EGFR and BIRC5 expression values were averaged for each subtype.

### Assessment of the effects of EGFR and BIRC5 expression on patient clinical parameters and outcomes

The 855-patient dataset was used to assess the relationships between EGFR/BIRC5 expression and clinical parameters/outcomes. Pearson correlations were performed to determine correlations between EGFR/BIRC5 expression and clinical characteristics (breast cancer intrinsic subtype, ER/PR/HER2 status, patient age, lymph node status, differentiation and proliferation scores, metastasis-free survival time, as well as relapse-free survival in the liver and lung). The 140 patients with basal-like tumors were ranked and divided based on EGFR and BIRC5 expression separately: EGFR^high^ (top 50%) or EGFR^low^ (bottom 50%) and BIRC5^high^ (top 50%) or BIRC5^low^ (bottom 50%). These patients were subsequently divided into four groups based on the designated expression levels (high or low) for each gene: EGFR^high^BIRC5^high^ (N = 32), EGFR^high^BIRC5^low^ (N = 38), EGFR^low^BIRC5^high^ (N = 38), EGFR^low^BIRC5^low^ (N = 32). Kaplan-Meier analysis was performed to determine the differences across these four groups in terms of metastasis-free survival time, liver relapse-free survival, and lung relapse-free survival.

### Statistical analyses

Statistical analyses were performed using unpaired student’s *t* tests to determine the significance of differences in cell viability between control and drug-treated conditions *in vitro*, as well as the significance between all treatment conditions *in vivo*; *p* < 0.05 was considered statistically significant. For the single-dose combination studies, we performed unpaired *t* tests to determine the significance of differences between mean differences in percent inhibition across PDXs, and we calculated 95% confidence intervals of the mean differences in percent inhibition and of the proportion of PDXs showing supra-additive or sub-additive trends bases on our analysis method. Where appropriate, data are presented as means ± standard deviations. Tukey’s multiple comparisons tests were performed to analyze differences in EGFR and BIRC5 expression between breast cancer subtypes using the 855-patient dataset. Relationships between EGFR and BIRC5 expression and clinical characteristics in the 855-patient dataset were analyzed by Pearson correlation. The effects of EGFR and BIRC5 expression on metastasis-free survival in patients with basal-like breast cancer were statistically analyzed using log-rank tests. All statistical tests were performed using GraphPad Prism 8.

## Supplementary information

Supplementary File S1.

Supplementary File S2.

Supplementary Info.

## Data Availability

The data generated and analyzed during this study are included in full or summarized format in this article. Data are available from the corresponding author on reasonable request.
