# Supplementary material for: Identification of synergistic drug combinations using breast cancer patient-derived xenografts
Source: Sci Rep. 2020 Jan 30;10:1493. doi: 10.1038/s41598-020-58438-0 (PMC6992640; doi:10.1038/s41598-020-58438-0)
Supplement: Supplementary file 1 — Supplementary File S1. [file 41598_2020_58438_MOESM1_ESM.pdf]

Supplementary Information

Identification of synergistic drug combinations using breast cancer patient-derived xenografts

Tia H. Turner[1,2], Mohammad A. Alzubi[1,3], and J. Chuck Harrell[1,2,3,4,*]

[1]Department of Pathology, Virginia Commonwealth University, Richmond, VA, USA

[2]Wright Center for Clinical and Translational Research, Virginia Commonwealth University, Richmond, VA, USA

[3]Integrative Life Sciences Doctoral Program, Virginia Commonwealth University, Richmond, VA, USA

[4]Massey Cancer Center, Virginia Commonwealth University, Richmond, VA, USA

Supplementary Table S1: P-values for *in vitro* dose response experiments shown in Figure 3. *t*-tests were performed to compare each drug treatment condition with vehicle controls for each PDX line. Significant values (p<0.05) are bolded and italicized.

**HCI01**	**N [a]**	**0.01μM**	**0.1μM**	**1μM**	**10μM**
Bortezomib	2	0.063381	***0.005474***	***0.000043***	***0.000092***
Carfilzomib	3	***0.04963***	***0.007833***	***0.005388***	***<0.000001***
Ixazomib	2	0.110962	0.06093	***0.001144***	***0.001801***
ABT-199	3	***0.007255***	***0.001172***	***0.00003***	***0.000083***
Birinapant	3	***0.000288***	***0.00015***	***0.000021***	***0.000003***
Embelin	3	***0.007419***	***0.000892***	***0.000018***	***0.001437***
Navitoclax	4	***0.001159***	***0.001534***	***0.000527***	***0.002758***
YM155	4	***0.000238***	***0.000005***	***<0.000001***	***<0.000001***
Abemaciclib	2	***0.014298***	***0.03196***	***0.000116***	***0.000001***
Afatinib	2	0.221783	***0.044243***	***0.036208***	N/A
Calcitriol	2	***0.000008***	***0.000193***	***0.002056***	***0.003609***
Dronedarone	2	***0.000991***	***0.002264***	***0.000811***	0.057112
Fluoxetine	2	***0.000008***	0.104927	***0.041811***	0.20296
**UCD52**	**N [a]**	**0.01μM**	**0.1μM**	**1μM**	**10μM**
Bortezomib	3	***0.026413***	***0.000039***	***<0.000001***	***<0.000001***
Carfilzomib	4	0.091647	***0.032814***	***<0.000001***	***<0.000001***
Ixazomib	3	***0.004423***	***0.014659***	***0.000068***	***<0.000001***
ABT-199	3	0.183095	0.652814	0.268487	***0.000003***
Birinapant	3	0.319556	***0.024892***	***0.006759***	***0.001068***
Embelin	3	0.156021	0.937212	0.868763	0.962227
Navitoclax	4	0.830949	0.996264	***0.029684***	***0.005872***
YM155	4	***0.000112***	***<0.000001***	***<0.000001***	***<0.000001***
Abemaciclib	4	0.29377	0.41761	***0.030087***	***<0.000001***
Afatinib	4	0.062199	***0.009366***	***0.000071***	***<0.000001***
Calcitriol	3	0.050349	***0.000418***	***0.000007***	***0.000643***
Dronedarone	3	0.317764	0.200828	***0.004828***	***0.000084***
Fluoxetine	3	0.16584	0.160174	0.073714	0.062933
**WHIM30**	**N [a]**	**0.01μM**	**0.1μM**	**1μM**	**10μM**
Bortezomib	3	0.815718	***0.000042***	***0.000006***	***<0.000001***
Carfilzomib	4	0.746654	***0.000106***	***<0.000001***	***0.000004***
Ixazomib	3	***0.000619***	0.361073	***0.000107***	***<0.000001***
ABT-199	2	0.301903	0.333421	0.534922	***0.014036***
Birinapant	2	***0.00193***	***0.004334***	***0.009629***	***0.011227***
Embelin	2	0.087373	0.168851	***0.031747***	0.114093
Navitoclax	2	***0.03891***	0.420622	0.490906	0.175765
YM155	2	***0.003486***	***0.000422***	***0.001594***	***0.000244***
Abemaciclib	2	0.686389	0.769689	0.97588	0.858871
Afatinib	2	0.208958	0.158912	0.378832	***0.001984***
Calcitriol	1	N/A	N/A	N/A	N/A

Table S1, *continued*

Dronedarone	1	N/A	N/A	N/A	N/A
Fluoxetine	1	N/A	N/A	N/A	N/A
**WHIM2**	**N** [a]	**0.01μM**	**0.1μM**	**1μM**	**10μM**
Bortezomib	3	0.068183	0.059901	0.058861	***0.000033***
Carfilzomib	4	0.310044	0.333459	***0.000359***	***0.000278***
Ixazomib	3	***0.000784***	0.05822	0.15067	0.06533
ABT-199	2	0.355539	0.089689	***0.028295***	0.669916
Birinapant	2	0.482637	***0.000351***	***0.002711***	***0.006058***
Embelin	2	0.572152	0.347313	0.115227	***0.040586***
Navitoclax	3	0.231318	***0.024578***	0.617452	***0.002852***
YM155	3	***0.000252***	***0.000069***	***0.001651***	***0.000426***
Abemaciclib	4	0.153799	0.459897	0.422449	***<0.000001***
Afatinib	4	0.167517	***0.003227***	***0.000542***	***0.000036***
Calcitriol	2	0.529639	***0.009585***	***0.002376***	***0.037265***
Dronedarone	2	0.534513	0.280651	0.977216	0.070761
Fluoxetine	2	0.175073	0.214316	0.663944	0.702763

[a] N indicates number of independent experiments for each drug tested.

Supplementary Table S2: Drug doses used for *in vitro* dose response experiments shown in Figure 4.

**Carboplatin (μM)**	**Dose 1**	**Dose 2**	**Dose 3**	**Dose 4**	**Dose 5**	**Dose 6**	**Dose 7**
HCI01	0.4	2	10	50	250	500	1000
UCD52	0.04	0.2	1	5	25	125	625
WHIM30	0.16	0.8	4	20	100	200	400
**Carfilzomib (μM)**	**Dose 1**	**Dose 2**	**Dose 3**	**Dose 4**	**Dose 5**	**Dose 6**	**Dose 7**
HCI01	0.004	0.02	0.1	0.5	2.5	12.5	62.5
UCD52	0.0016	0.008	0.04	0.2	1	5	25
WHIM30	0.0008	0.004	0.02	0.1	0.5	2.5	12.5
**Afatinib (μM)**	**Dose 1**	**Dose 2**	**Dose 3**	**Dose 4**	**Dose 5**	**Dose 6**	**Dose 7**
HCI01	0.004	0.02	0.1	0.5	2.5	12.5	62.5
UCD52	0.0004	0.002	0.01	0.05	0.25	1.25	6.25
WHIM30	0.04	0.2	1	5	10	20	40
WHIM2	0.0004	0.002	0.01	0.05	0.25	1.25	6.25
**YM155 (μM)**	**Dose 1**	**Dose 2**	**Dose 3**	**Dose 4**	**Dose 5**	**Dose 6**	**Dose 7**
HCI01	0.00008	0.0004	0.002	0.01	0.05	0.25	1.25
UCD52	0.00004	0.0002	0.001	0.005	0.025	0.125	0.625
WHIM30	0.00004	0.0002	0.001	0.005	0.025	0.125	0.625
WHIM2	0.00008	0.0004	0.002	0.01	0.05	0.25	1.25

Supplementary Table S3: P-values for *in vitro* dose response experiments shown in Figure 4. *t*-tests were performed to compare each drug treatment condition with vehicle controls for each PDX line. Significant values (p<0.05) are bolded and italicized.

**Carboplatin**	**Dose 1**	**Dose 2**	**Dose 3**	**Dose 4**	**Dose 5**	**Dose 6**	**Dose 7**
HCI01	***0.011969***	0.188374	***0.000454***	***0.003011***	***0.000032***	***0.000084***	***0.000222***
UCD52	0.073298	***0.018515***	0.108632	***0.024821***	***0.000717***	***0.000519***	***0.000599***
WHIM30	***0.046447***	***0.000012***	0.090614	***0.008275***	***0.000309***	***<0.000001***	0.063431
**Carfilzomib**	**Dose 1**	**Dose 2**	**Dose 3**	**Dose 4**	**Dose 5**	**Dose 6**	**Dose 7**
HCI01	0.425251	***0.007535***	***0.013284***	***0.001064***	***0.000463***	***0.000027***	***0.000004***
UCD52	***0.000417***	0.291974	***0.023256***	***0.017735***	***0.000731***	***0.000625***	***0.000382***
WHIM30	***0.00319***	0.131364	***0.000132***	***0.0323***	***0.000143***	***0.000083***	***<0.000001***
**Afatinib**	**Dose 1**	**Dose 2**	**Dose 3**	**Dose 4**	**Dose 5**	**Dose 6**	**Dose 7**
HCI01	0.076782	0.210436	***0.001735***	***0.007566***	***0.000711***	***0.000058***	***0.000073***
UCD52	0.210197	***0.007077***	***0.009177***	***0.002972***	***0.019954***	***0.000267***	***0.000522***
WHIM30	***0.00052***	***0.028389***	***0.013618***	***0.000322***	***0.000013***	***0.000003***	***0.000002***
WHIM2	0.187042	0.99756	0.257375	***0.012296***	***0.001094***	0.115623	***0.000009***
**YM155**	**Dose 1**	**Dose 2**	**Dose 3**	**Dose 4**	**Dose 5**	**Dose 6**	**Dose 7**
HCI01	***0.000167***	0.154071	***0.011395***	***0.016656***	***0.00195***	***0.000156***	***0.000009***
UCD52	0.217038	0.099011	***0.009378***	***0.047491***	***0.005413***	***0.001095***	***0.000506***
WHIM30	0.18806	0.088101	0.08598	***0.000325***	***0.000149***	***0.000179***	***0.000579***
WHIM2	0.994948	***0.00007***	***0.032879***	0.572891	***0.001695***	***0.000122***	***0.000029***

Supplementary Table S4: P-values for *in vitro* dose response experiments shown in Figure 7a-b. *t*-tests were performed to compare each drug treatment condition with vehicle controls for each cell line. Significant values (p<0.05) are bolded and italicized.

**Afatinib**	**0.0032µM**	**0.016µM**	**0.08µM**	**0.4µM**	**2µM**	**10µM**	**50µM**
MDA468	***0.005145***	***0.00005***	***0.000268***	***0.000776***	***0.000685***	***0.005603***	***0.000036***
HCC1143	***0.003195***	0.107024	***0.048012***	***0.004614***	***0.018885***	***0.016815***	***0.000443***
HCC1937	***0.028664***	***0.007818***	***0.015839***	***0.000845***	***0.014367***	***0.003326***	***0.001422***
**YM155**	**0.000064µM**	**0.00032µM**	**0.0016µM**	**0.008µM**	**0.04µM**	**0.2µM**	**1µM**
MDA468	***0.046448***	***0.041252***	***0.005506***	***0.000008***	***<0.000001***	***<0.000001***	***<0.000001***
HCC1143	***0.002632***	***0.008782***	***0.026343***	***0.012459***	***0.000029***	***0.000006***	***<0.000001***
HCC1937	***0.015797***	***0.003415***	***0.0013***	***0.001102***	***0.000002***	***0.000008***	***<0.000001***

Supplementary Table S5: P-values for *in vivo* drug treatment experiments shown in Figure 8a,c. *t*-tests were performed to compare all treatment conditions at each timepoint for tumor growth and at the study endpoint for tumor weights. Significant values ($p<0.05$) are bolded and italicized.

**Treatment group comparison**	**Tumor growth**					**Tumor weights**
	**Day 14**	**Day 17**	**Day 21**	**Day 24**	**Day 28**	**Endpoint**
Untreated vs. Afatinib	>0.999999	0.101192	0.06405	0.089521	***0.015093***	0.212763
Untreated vs. YM155	>0.999999	0.106166	***0.003654***	***0.001496***	***0.001343***	***0.000315***
Untreated vs. Afatinib+YM155	>0.999999	***0.017797***	***0.002192***	***0.000991***	***0.001174***	***0.000157***
Afatinib vs. YM155	>0.999999	0.724659	0.125798	0.098082	***0.01975***	0.067574
Afatinib vs. Afatinib+YM155	>0.999999	0.237796	0.054921	0.054978	***0.012391***	0.054311
YM155 vs. Afatinib+YM155	>0.999999	0.565533	0.101192	0.228229	0.403088	0.441823

Supplementary Table S6: P-values for Kaplan-Meier survival curves shown in Figure 12. Log-rank tests were performed between all basal-like patient groups (based on EGFR/BIRC5 expression levels) for relapse-free survival (liver, and lung) and metastasis-free survival (MFS) time. Significant values ($p<0.05$) are bolded and italicized.

**Liver relapse**			**P-value**
$EGFR^{high}/BIRC5^{high}$	vs	$EGFR^{high}/BIRC5^{low}$	0.0764
$EGFR^{high}/BIRC5^{high}$	vs	$EGFR^{low}/BIRC5^{high}$	***0.0153***
$EGFR^{high}/BIRC5^{high}$	vs	$EGFR^{low}/BIRC5^{low}$	0.0787
$EGFR^{high}/BIRC5^{low}$	vs	$EGFR^{low}/BIRC5^{high}$	0.627
$EGFR^{high}/BIRC5^{low}$	vs	$EGFR^{low}/BIRC5^{low}$	0.9262
$EGFR^{low}/BIRC5^{high}$	vs	$EGFR^{low}/BIRC5^{low}$	0.8038
**Lung relapse**			**P-value**
$EGFR^{high}/BIRC5^{high}$	vs	$EGFR^{high}/BIRC5^{low}$	***0.0284***
$EGFR^{high}/BIRC5^{high}$	vs	$EGFR^{low}/BIRC5^{high}$	***0.0204***
$EGFR^{high}/BIRC5^{high}$	vs	$EGFR^{low}/BIRC5^{low}$	***0.0337***
$EGFR^{high}/BIRC5^{low}$	vs	$EGFR^{low}/BIRC5^{high}$	0.9187
$EGFR^{high}/BIRC5^{low}$	vs	$EGFR^{low}/BIRC5^{low}$	0.9291
$EGFR^{low}/BIRC5^{high}$	vs	$EGFR^{low}/BIRC5^{low}$	0.8673
**MFS time**			**P-value**
$EGFR^{high}/BIRC5^{high}$	vs	$EGFR^{high}/BIRC5^{low}$	***0.022***
$EGFR^{high}/BIRC5^{high}$	vs	$EGFR^{low}/BIRC5^{high}$	***0.0178***
$EGFR^{high}/BIRC5^{high}$	vs	$EGFR^{low}/BIRC5^{low}$	***0.009***
$EGFR^{high}/BIRC5^{low}$	vs	$EGFR^{low}/BIRC5^{high}$	0.8503
$EGFR^{high}/BIRC5^{low}$	vs	$EGFR^{low}/BIRC5^{low}$	0.42
$EGFR^{low}/BIRC5^{high}$	vs	$EGFR^{low}/BIRC5^{low}$	0.213

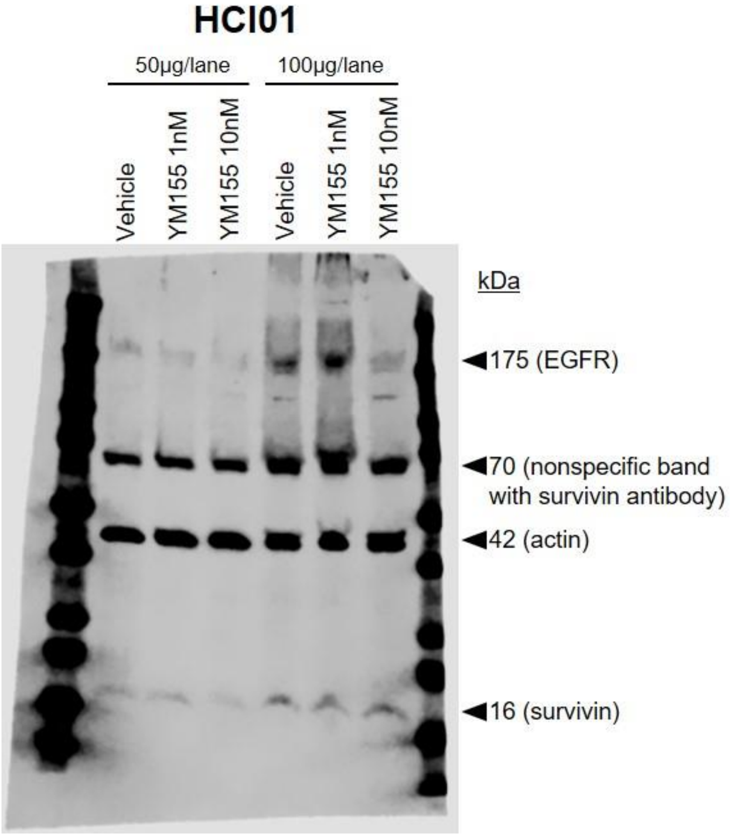

Supplementary Figure S1: Uncropped Western blot associated with Figure 9a-b, in which EGFR and actin of the 100µg/lane samples were depicted and used for densitometry analysis.
